# A genome-wide scan shows evidence for local adaptation in a widespread keystone Neotropical forest tree

**DOI:** 10.1038/s41437-019-0188-0

**Published:** 2019-02-12

**Authors:** Rosane G. Collevatti, Evandro Novaes, Orzenil B. Silva-Junior, Lucas D. Vieira, Matheus S. Lima-Ribeiro, Dario Grattapaglia

**Affiliations:** 1 Laboratório de Genética & Biodiversidade, Instituto de Ciências Biológicas, Universidade Federal de Goiás, Goiânia, GO 74001-970 Brazil; 2 Departamento de Biologia, Universidade Federal de Lavras, Lavras, MG 37200-000 Brazil; 3 EMBRAPA Recursos Genéticos e Biotecnologia, EPqB, Brasília, DF 70770-910 Brazil; 4 Programa de Ciências Genômicas e Biotecnologia–Universidade Católica de Brasília, SGAN 916 Modulo B, Brasilia, DF 70790-160 Brazil; 5 Laboratório de Macroecologia, Universidade Federal de Goiás (UFG), Campus Jataí, Jataí, GO 75801-615 Brazil

**Keywords:** Genetic variation, Conservation genomics

## Abstract

The role of natural selection in shaping patterns of diversity is still poorly understood in the Neotropics. We carried out the first genome-wide population genomics study in a Neotropical tree, *Handroanthus impetiginosus* (Bignoniaceae), sampling 75,838 SNPs by sequence capture in 128 individuals across 13 populations. We found evidences for local adaptation using Bayesian correlations of allele frequency and environmental variables (32 loci in 27 genes) complemented by an analysis of selective sweeps and genetic hitchhiking events using SweepFinder2 (81 loci in 47 genes). Fifteen genes were identified by both approaches. By accounting for population genetic structure, we also found 14 loci with selection signal in a STRUCTURE-defined lineage comprising individuals from five populations, using Outflank. All approaches pinpointed highly diverse and structurally conserved genes affecting plant development and primary metabolic processes. Spatial interpolation forecasted differences in the expected allele frequencies at loci under selection over time, suggesting that *H. impetiginosus* may track its habitat during climate changes. However, local adaptation through natural selection may also take place, allowing species persistence due to niche evolution. A high genetic differentiation was seen among the *H. impetiginosus* populations, which, together with the limited power of the experiment, constrains the improved detection of other types of soft selective forces, such as background, balanced, and purifying selection. Small differences in allele frequency distribution among widespread populations and the low number of loci with detectable adaptive sweeps advocate for a polygenic model of adaptation involving a potentially large number of small genome-wide effects.

## Introduction

The investigation of the relative contributions of demography and natural selection to the spatial patterns of genetic variation has been a recurring theme in evolutionary biology. Spatial variations in the pattern of natural selection can lead to local adaptation and genetic differentiation among populations. Adaptation to spatially varying selective pressures is evident in the geographical distribution of many traits in plants (e.g., Linhart and Grant, 1996; Joshi et al. 2001; Sakai and Larcher, 2012). However, the role of natural selection in shaping the patterns of plant diversity and adaptation is still poorly understood (Savolainen et al. 2013; Tiffin and Ross-Ibarra, 2014), particularly in the tropics, where the greatest diversity of plant species is found.

With increasingly more powerful and accessible DNA technologies, genome scans have allowed the scrutinising of candidate genomic regions for signals of local adaptation. This approach is possible because adaptation tends to shape the pattern of genetic variation within and between loci (e.g., Maynard-Smith and Haigh, 1974; Kaplan et al. 1989). Selection footprints may be detected depending on the number of generations since selection, the strength of the selection sweep, and the amount of recombination. However, caution is necessary as many demographical events can result in similar patterns of polymorphism (Hohenlohe et al. 2010; Nei et al. 2010).

Forest trees have become an interesting experimental target for population, evolutionary, and ecological genomic investigations in an attempt to understand the molecular basis of local adaptation (Lind et al. 2018). Most adaptive traits in forest trees such as stem growth, environmental tolerances, and pest and disease resistance are usually under polygenic control, and much has been learned about their inheritance using quantitative genetics methods applied to common garden experiments (White et al. 2007). Even though quantitative genetics methods provide estimates of heritability, they cannot inform about the location and relative contributions of individual genes or genomic regions to the adaptive trait under scrutiny.

Genome-wide identification of adaptation signals in forest trees is a challenging task but advances have been made in the past 10 years mostly in those few forest tree species of economic relevance for which genomic resources have been developed (Kremer et al. 2011; Savolainen et al. 2013; Neale and Kremer, 2011). The dropping costs and increasing data yields of next-generation sequencing technologies have fostered the development of a number of sequencing-based genotyping methods that allow the simultaneous discovery and genotyping of very large numbers of markers (Davey et al. 2011). Among the several methods available today, targeted enrichment or sequence capture (Mamanova et al. 2010) has been increasingly used in forest trees such as pines and spruces (Neves et al. 2013; Syring et al. 2016; Yeaman et al. 2016) and Poplars (Zhou and Holliday, 2012). This methodology significantly reduces costs and effort compared with whole-genome sequencing because only specific loci of interest are captured and sequenced at high-depth coverage, increasing single-nucleotide polymorphism (SNP)-genotyping confidence. The practical advantages and increasing accessibility of target sequence-capture methods have been reviewed in the context of evolutionary and ecological genomics, predicting a rapid expansion of this approach to address fundamental biological questions at a scale that was unimaginable just a few years ago (Jones and Good, 2016).

Genome scans have detected signatures of selection and associations with adaptive traits using several thousand SNPs in candidate genes of *Populus* (McKown et al. 2014) and DArT markers that targeted the gene space in *Eucalyptus* (Steane et al. 2014). In *Populus*, whole-genome sequencing revealed hundreds of genomic regions showing evidence of recent positive and/or divergent selection, as well as enrichment for associations with adaptive traits that displayed patterns consistent with natural selection (Evans et al. 2014). In conifers, despite their challenging mega-genomes, extensive genome scans have been reported, targeting up to ~7000 candidate genes in spruces (*Picea* sp.) (Hornoy et al. 2015) and sampling over a million SNPs in 23,000 genes in two distantly related conifers, lodgepole pine and interior spruce (Yeaman et al. 2016). No such genome-wide study has been reported yet for Neotropical plant species, mostly due to the lack of genome resources for efficient SNP discovery and genotyping (Pool et al. 2010).

Besides perenniality, forest trees display high levels of genetic and phenotypic variation across large areas covering variable environments, frequently showing latitudinal clines in the timing of growth cessation or initiation in temperate zones (Savolainen et al. 2007; Grattapaglia et al. 2009; Neale and Kremer, 2011). Furthermore, fossil records of tree species show range shifts in response to Quaternary climate changes (e.g. Kremer and Goenaga, 2002; Magri et al. 2006), and shifts in geographical range are also evidenced in phylogeographical analyses in both temperate (e.g. Petit et al. 2002) and tropical species (e.g. Collevatti et al. 2015a). Changes in palaeodistribution and demography associated with climate changes point to niche conservatism rather than adaptation to new environmental conditions—i.e. the “Habitat (or niche) Tracking” hypothesis (Parmesan and Yohe, 2003; Eldredge et al. 2005). However, evidences based on molecular and quantitative data, at least in temperate species, suggest that adaptation after postglacial colonisation is the predominant factor that shapes the present quantitative-trait variation in temperate zones (e.g. Kremer et al. 2002). These two contrasting patterns raise the question whether the distribution of genetic diversity in the Neotropics is driven more by natural selection or by demographical history. The reconstruction of species past distributions based on the simulation of independent palaeoscenarios of demographical history using coalescence analysis (e.g., Carstens and Richards, 2007; Collevatti et al. 2012, 2013) provides a valuable tool to understand the role of demographical history in the current distribution of genetic diversity (Collevatti et al. 2015b). Therefore, a framework coupling coalescent models with genome scans may provide clues on the different roles of natural selection and genetic drift in species evolution.

*Handroanthus impetiginosus* (Mart. ex DC.) Mattos *(*syn*. Tabebuia impetiginosa*, Bignoniaceae) is a Neotropical hardwood tree with a widespread distribution throughout the seasonally dry tropical forests (SDTFs) of South and Mesoamerica. It ranges from Northeast to Southwestern Brazil, Bolivia, and is also scattered throughout the fragments of SDTFs in Central Brazil and on the slopes of Andes in Peru (Collevatti et al. 2012). It is a highly valued timber species regarded as the “new mahogany” for its dense (0.96 g/cm^3^), rot-resistant wood. It is the second most expensive timber and the most logged species in Brazil (Schulze et al. 2008), exported largely to North America for residential decking and currently under significant illegal trading pressure. *H. impetiginosus* is a diploid species (2*n* = 40) with a genome size estimated at 2C *c*. 1Gb by flow cytometry (Collevatti and Dornelas, 2016). More recently, we have sequenced and assembled the genome of *H. impetiginosus*, predicting and annotating 31,688 genes (Silva-Junior et al. 2018a). The genome assembly covered 503.7 Mb (N50 = 81,316 bp), 90.4% of the 557 Mb genome, with 13,206 scaffolds.

Previously, we have investigated the phylogeography and demographical history of *H. impetiginosus*, sampling 17 populations in Brazil, based on hindcasting species-distribution modelling and polymorphism in the chloroplast genome and nuclear nuclear ribosomal DNA (nrDNA) internal transcribed spacer (ITS) (Collevatti et al. 2012). Populations showed high levels of genetic differentiation but with incomplete lineage sorting most likely due to palaeodistribution expansion during the last glacial maximum (LGM). The high differentiation among populations, coupled with the widely disjunct distribution, suggests an initial hypothesis that adaptive selection may be shaping the current patterns and distribution of genetic variation in *H. impetiginosus*. On the other hand, the demographical changes over time may have led to the low population effective size and the associated limited gene flow (Collevatti et al. 2012), suggesting that this drift is likely responsible for the high genetic differentiation observed among populations (Kimura, 1991). A genome-wide scan of molecular diversity may, therefore, allow a comprehensive evaluation of these alternative hypotheses, providing an increased understanding of the evolutionary processes underlying the genetic diversity and population differentiation observed in *H. impetiginosus*. An improved knowledge of the evolutionary history and population genomics of this highly exploited species may also help define management units for conservation planning (Shafer et al. 2015) and provide some initial clues on the adaptive mechanisms of tropical forest trees to variable environments.

In this study, we carried out a population genomics survey of *H. impetiginosus* based on a capture-based target-enrichment system (Silva-Junior et al. 2018b) targeting 10,246 loci across the genome of *H. impetiginosus* (Silva-Junior et al. 2018a). With this system, 75,838 SNPs were genotyped in 128 individuals across 13 populations. Different approaches were used in an attempt to detect selection sweeps, including outlier detection, correlation of allele frequencies with environmental variables, and likelihood ratio tests to detect selective sweeps and genetic hitchhiking. Additionally, spatial interpolation (co-kriging) of candidate loci under selection was performed to understand the expected allele frequency across the SDTFs for the present day, LGM (21 ka), and the end of century (EOC). Finally, coalescent simulations were used to reconstruct the demographical history of the species in an attempt to disentangle the effects of demography and natural selection on the distribution of genetic diversity.

## Materials and methods

### Population sampling

**Fig. 1:**
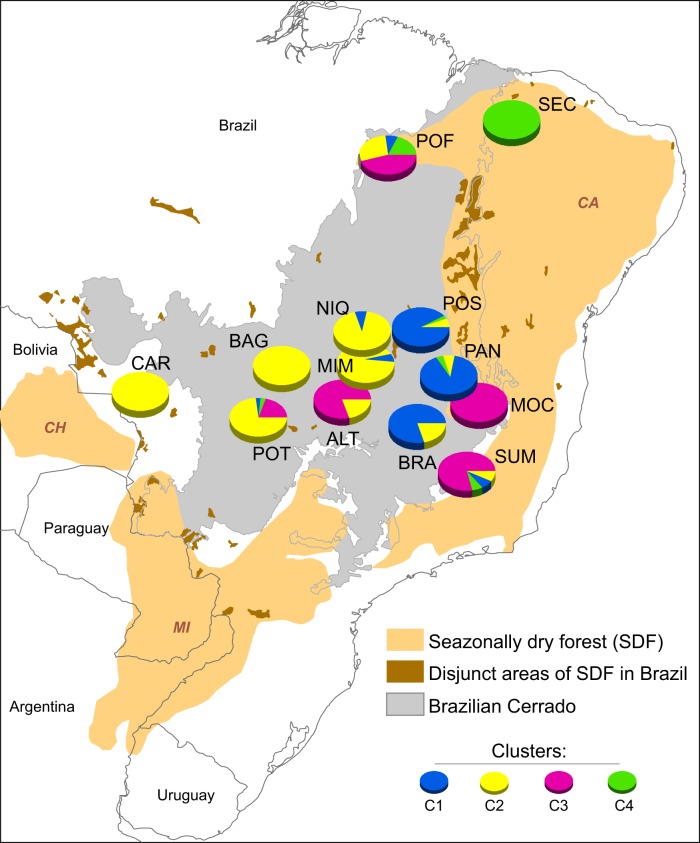
Geographical distribution of the 13 *Handroanthus impetiginosus* populations sampled for genetic analyses and the respective Bayesian clustering of individuals based on 200 putatively neutral SNP loci. Each colour represents the inferred cluster (*K* = 4) following the figure legends. The size of cluster chart section represents population coancestry for each cluster. Details on the sampled populations are provided in Supplementary File S1 and Table S1

We sampled 13 populations with an average of 9.8 individuals per population, totalling 128 individuals, throughout the geographical distribution of *H*. *impetiginosus* in Brazil (Fig. 1; Supplementary File S1 Table S1). Adult individuals were Global Positioning System (GPS) mapped, and their expanded leaves were sampled for DNA extraction, which was carried out using Qiagen DNeasy Plant Mini kit (Qiagen, DK).

### SNP genotyping

A set of 14,135 120-mer probe sequences targeting 11,026 distinct loci of the 30,271 predicted protein-coding genes across the genome assembly of *H. impetiginosus* (Silva-Junior et al. 2018a, b) was used for DNA target enrichment to direct genotype SNPs in 128 individual trees. Targeted DNA enrichment, capture and sequencing were carried out as described earlier (Silva-Junior et al. 2018b). Sequence data analysis and screening for polymorphism were performed with GATK-HaplotypeCaller (McKenna et al. 2010) after all reads were aligned to the whole-genome assembly of *H. impetiginosus* with BWA software (Li and Durbin, 2009). A more detailed description of the sequence capture and SNP-genotyping pipeline is provided in Supplementary File S1.

### Genome-wide diversity and genetic structure

To characterise the genome-wide diversity, we calculated the density of SNPs across all probes using a bin size of 10,000 bp, the allele frequencies, and the percentage of missing data. We also obtained the ratio of transition to transversion substitutions (Ts/Tv) and expected heterozygosity (Nei, 1987). These parameters were estimated using VCFtools (Danecek et al. 2011).

To characterise the population genetic diversity, we estimated the expected heterozygosity under Hardy–Weinberg equilibrium (He) and inbreeding coefficient (*f*) using Arlequin 3.5 (Excoffier et al. 2005). We also estimated the genetic differentiation among populations (*F*_ST_) and inbreeding coefficient (*F*_IS_), across all SNPs, using analysis of molecular variance implemented in Arlequin 3.5 (Excoffier et al. 2005). Significance levels of 0.05 for each estimate were determined with 10,000 permutations.

To better understand the different roles of selection and genetic drift in the evolution of *H. impetiginosus*, we estimated the genetic diversity and population structure using only putatively neutral loci, i.e. excluding loci that might carry selection footprints. For that, we randomly selected 200 loci from those displaying a Bayenv2 Bayes Factor (BF) < 0.1 (see results below) in different genome scaffolds to minimise linkage disequilibrium. We also accessed the genetic structure of the populations using the 200 putatively neutral loci to obtain the most likely number of genetic clusters (Supplementary File S1) using Structure 2.3.4 (Pritchard et al. 2000).

### Demographical history simulation

The past demographical history of *H. impetiginosus* to the present day was modelled and simulated based on coalescent analysis implemented in the software fastsimcoal25 (Excoffier and Foll, 2011; Excoffier et al. 2013). We modelled four demographical scenarios following the framework described in Collevatti et al. (2012, 2013). For each demographical scenario, we ran 2000 independent simulations for 200 putatively neutral SNPs (Supplementary File S1). The number of generations until the LGM was calculated using a generation time of 15 years (Collevatti et al. 2012). Demographical hypotheses were simulated backward, with 13 demes from time t0 (present) to t1400 generations ago (at the LGM) using the same parameterisation described in Collevatti et al. (2012). Effective population sizes at N0 = 10,000 (total effective population size at present) were the same for all scenarios, and N1400 (effective population size 1400 generations ago, at the LGM) varied among the hypotheses according to the theoretical expectation. Simulated alternative models were compared based on the distribution of expected heterozygosity in the simulations using two-tailed probabilities and Akaike information criterion (AIC) for model choice.

### Genome scans for selection footprints

We used three genome scan approaches in an attempt to detect the loci under selection. First, we used the approach implemented by Outflank (Whitlock and Lotterhos, 2015) to detect local adaptation based on the expected distribution of *F*_ST._ Other methods based on *F*_ST_ outliers usually assume a specific model of demographical history, which can result in higher false-positive rates. In the absence of selection, *F*_ST_ is expected to have a Chi-square distribution. We performed two analyses. First, we used the entire SNP dataset. Second, we selected only one SNP locus per probe to minimise linkage disequilibrium. SNPs for which all individuals were heterozygous were also removed, with 10,340 loci remaining. To generate the distributions, we trimmed the *F*_ST_ distribution at 5 and 10% and used a minimum expected heterozygosity of 0.10 and a false discovery rate < 0.05.

We then used a Bayesian framework implemented in Bayenv2 (Coop et al. 2010; Günther and Coop, 2013) that looks for local adaptation by estimating linear correlations between allele frequencies and environmental variables, while controlling for relationships among populations. Four bioclimatic variables from the WorldClim Global Climate Bioclim database (www.worldclim.org/bioclim) with a spatial resolution of 30” (0.93 × 0.93 = 0.86 km^2^ at the equator) were obtained for the 13 populations sampled. We performed a factorial analysis with Varimax rotation and selected four variables with low collinearity. These four variables explain 90.4% of the total environmental variation among the 13 populations (Supplementary File S1 and Table S2). The selected variables were Bio4 (temperature seasonality), Bio5 (maximum temperature of the warmest month), Bio16 (precipitation of the wettest quarter) and Bio17 (precipitation of the driest quarter). We also obtained subsoil (30–100 cm) data related to soil fertility from the Harmonized World Soil Database (version 1.2, FAO/IIASA/ISRIC/ISS-CAS/JRC 2009, available at http://www.fao.org/docrep/018/aq361e/aq361e.pdf). Using Varimax factorial analysis, we selected three soil variables that explained 77.5% of the variation among populations (Supplementary File S1 and Table S3): clay cationic exchange capacity (CEC Clay), CaCO_3_ concentration, and base saturation (BS). Loci with adaptive selection signal were selected with the following criteria, following the directions of Günther and Coop (2013): a high Bayes Factor (BF > 10), i.e. the ratio of the likelihood probability of the hypothesis of linear relationship (between allele frequency and environmental variable) and the null hypothesis (no linear relationship) given the data, and a correlation coefficient (Spearman’s correlation) above |0.15|. These values were in the 0.1% quantile of the distribution of the data.

Finally, we used the software SweepFinder2 v. 1.0 (DeGiorgio et al. 2016) to detect selective sweeps and genetic hitchhiking based on deviations of a neutral null hypothesis. We applied the composite likelihood ratio test only to 27 scaffolds > 10,000 bp. With the objective of identifying the possible location of recent selective sweeps in each population, we followed Nielsen et al. (2005), and a detailed description of the analytical procedure and parameters used is provided in Supplementary File S1.

Putative SNP loci under selection were annotated with SNPEff (Cingolani et al. 2012) with respect to the annotation in the reference genome (Silva-Junior et al. 2018a). If an SNP was annotated as being in the coding region of a gene and upstream of another, only the coding-sequence annotation was kept. Affected genes were analysed with respect to their functional annotation in terms of Gene Ontology (GO) categories (Ashburner et al. 2000). GO enrichment analysis was performed on the dataset of genes closest to the loci deemed to be under selection. Analysis of the binary association of the ontology terms of the genes within the region near (10-kb upstream/downstream) to the sweep (‘1’ value) and distant (‘0’ value) from it was performed using the program func_hyper in the FUNC package (Prüfer et al. 2017). Significance of the enrichment was assessed in terms of approximated *p* values derived using a sensitive estimator of the global significance of the test results computed for subtrees of each top ontology category (biological process, molecular function and cellular components).

### Spatial prediction and shifts in allele frequency

For the loci under selection, we applied co-kriging to the Neotropics using ArcGIS 10.2 to obtain a spatial interpolation of allele-frequency estimates based on their autocorrelation with the climatic and soil variables. We also hindcasted the allele frequency for loci under selection in the LGM and forecast how future climate changes might affect the distribution in allele frequencies. We obtained the same environmental variables used in Bayenv2 analysis (Bio4, Bio5, Bio16, and Bio17) for the LGM from the Coupled Model Intercomparison Project Phase 5 (CMIP5) database (http://cmippcmdi.llnl.gov/cmip5/) and for the EOC (2100) using the RCP 4.5 (rising radioactive forcing pathway leading to 4.5 W/m^2^ in 2100) from the RCP database v 2.0.5 (http://tntcat.iiasa.ac.at/RcpDb). We assumed the soil variables constant through time.

We then calculated and plotted the number of shifts in allele frequency for the loci under selection. Shift was defined as the difference between the allele frequency of the population of the highest and the lowest value for each climatic and soil variable and for each SNP locus. For each variable, we obtained a vector of differences in allele frequency for the loci and then we calculated the density function using the function *density* implemented in the *stat* package in R 3.6.0.

## Results

### Sequence capture and SNP detection and genotyping

Sequence capture with 14,135 sequence probes targeted 11,026 distinct loci across the genome assembly generating a VCF file with 304,488 SNPs. After the removal of sites with more than 20% missing data, a total of 75,838 high-quality polymorphic SNPs with a call rate > 88.3% were detected. The average per-sample call rate across all 128 individuals was 93.4%. The *H. impetiginosus* SNPs revealed a ratio of transitions to transversions (Ts/Tv) of 2.02 and an average missing genotype of only 6.6% in the sample of 128 trees. The distribution of minor allele frequency (MAF) showed the expected “L” shape distribution with a larger proportion of low-frequency SNPs. A vast majority of SNPs (75.1%) had an MAF < 0.15, and the median MAF was equal to 0.071. These SNPs were found in coordinates of 4,862 distinct targeted loci across 2,341 scaffolds in the genome assembly. The resulting average read depth at SNP coordinates across the samples was 69.6 x ± 41.7 x, and the minimum depth of aligned reads to call a heterozygous genotype for a sample was 3 with the median equal to 14. Coordinates of these loci are within or in close proximity (≤ 5kb upstream/downstream of transcription initiation) to 6,489 of the 30,271 predicted protein-coding genes.

### Genome-wide diversity and genetic structure

We estimated the density of SNPs across the genome assembly, partitioned in 10,802 virtual bins of 10kb. As expected for sequence capture-based SNP genotyping, a large proportion (63.5% or 6858 regions) of the bins had no SNP identified in the sampled individuals. The average density of SNPs (Supplementary File S2 and Table S1), using a bin size of 10kb, was 7.02 SNPs/10 kb (SD = 13.11 SNPs/10kb, median = 0.0, min = 0.0, max = 153).

Thirteen populations showed significant genetic differentiation for the 75,838 SNPs (*F*_ST_ = 0.513, *p* < 0.001) and no significant inbreeding (*F*_IS_ = 0.031, *p* = 0.227). Populations POF, ALT, and MOC had the highest genetic diversity (He) over all loci (Supplementary File S1 and Table S4). Only population POT had a significant and high inbreeding coefficient (*f* = 0.566; Supplementary File S1 and Table S4). The total genetic diversity considering all the 75,838 SNPs (He = 0.169, SD = 0.138) did not differ from the genetic diversity calculated based only on the putatively neutral loci selected based on Bayenv2 results (He = 0.162, SD = 0.129). Again, only the population POT showed significant inbreeding for neutral loci. We also found a similar genetic differentiation (*F*_ST_ = 0.528, *p* < 0.001) when using only putatively neutral loci. Bayesian clustering supported four independent genetic clusters (*K* = 4), showing high genetic differentiation, but with admixture in some populations (Fig. 1; Supplementary File S3 and Fig. S1; Supplementary File S1 and Table S5).

### Simulation of demographical history

The scenario “Both” (an expansion throughout the Central and Southwest Brazil and also towards the interior of Amazon Basin) was the most likely model among the four tested to predict the observed genetic parameters of *H. impetiginosus*, using either two-tailed probability or AICw criterion (Supplementary File S1 and Table S6). However, the scenario Pleistocene Arc (i.e., an expansion throughout the Central and Southwest Brazil) could not be rejected (ΔAIC = 1.25). Nonetheless, both scenarios indicate a range expansion during the LGM, followed by a retraction towards the present day.

### Genome scan for selection footprints

The genome scan based on Outflank detected no outlier loci (Supplementary File S3 and Fig. S2; Supplementary File S2 and Table S2). As genome-wide differentiation among *H. impetiginosus* populations was high (*F*_ST_ = 0.513), the threshold for the detection of selection signals was also high. Because of the high differentiation among populations, we performed an additional genome-scan analysis using Outflank for individuals belonging to cluster C2, the only cluster with a sufficient sample size to warrant acceptable statistical power for the analysis. Cluster C2 included 34 individuals from populations BAG, CAC, MIM, NIQ, and POT (Fig. 1), which showed significant, but considerably lower, differentiation (*F*_ST_ = 0.074, *p* < 0.001). Using only samples from these populations, Outflank found 14 loci with the selection signal. These outlier loci had *F*_ST_ values ranging from 0.63 to 0.73.

Analysis with Bayenv2 identified a few loci with a high BF for both climatic (Supplementary File S3 and Fig. S3) and soil variables (Supplementary File S3 and Fig. S4). In addition, correlation coefficients were low, ranging between + 0.20 and - 2 (Figs. S3 and S4). For both climatic and soil variables, we declared loci with putative adaptive selection signal in those with BF > 10.0 and Spearman correlation |*ρ*| > 0.15 (Tables 1 and 2). For climatic variables, 21 loci were selected (Table 1). Eleven loci showed a high BF with soil variables, but their correlation coefficients were lower than those for climatic variables (Table 2).

**Table 1:** SNPs with potential for adaptive selection based on Bayes factor for bioclimatic variables (BF > 10 for at least one bioclimatic variable) and correlation (Spearman’s correlation, |*ρ*| > 0.15) using Bayenv2 software

Class	Genbank accession	SNP position	BF Bio04	*ρ* Bio04	BF Bio05	*ρ* Bio05	BF Bio16	*ρ* Bio16	BF Bio17	*ρ* Bio17	Affected gene	Annotation	Function and SNPEff annotation
D	NKXS01007262	727	**1.20E** **+191**	0.16981	**1.48E** **+** **130**	-0.1249	**2.41E** **+** **200**	-0.2012	**3.07E** **+** **21**	-0.0519			intergenic_region|MODIFIER
D-	NKXS01007262-	754	**4.15E** **+** **123**	0.13618	**4.10E** **+** **75**	-0.0941	**1.78E** **+** **170**	-0.1606	**3.59E** **+** **15**	-0.0982	Haimp10041442m.g	PF00006	ATP synthase alpha/beta family, nucleotide-binding domainupstream_gene_variant|MODIFIER-
A	NKXS01005069	42043	**983440000.0**	-0.19315	**5047700.0**	0.1315	**2818.80**	0.1489	0.0803	0.0218	Haimp10032484m.g	GO:0008270	zinc ion binding downstream_gene_variant|MODIFIER
A	NKXS01000550	14775	**1257900.0**	-0.15949	**11.9500**	0.0974	**21362000.0**	0.1447	0.1058	0.0371	Haimp10033596m.g	GO:0007094	mitotic spindle assembly checkpoint intron_variant|MODIFIER
C	NKXS01007262	851	**110.9500**	0.12024	3.6660	-0.1267	**402.880**	-0.1650	0.1148	0.0224	Haimp10041442m.g	PF00006	ATP synthase alpha/beta family, nucleotide-binding domainsynonymous_variant|LOW-
E	NKXS01009478	3168	**85.7210**	-0.15862	4.6344	0.0933	8.5058	0.1298	0.1482	0.0078	Haimp10004827m.g	KOG2451	Aldehyde dehydrogenase synonymous_variant|LOW
F	NKXS01008263	19989	2.5250	-0.14615	0.7613	0.1759	**17.8370**	0.1505	0.0619	-0.0827	Haimp10001090m.g	GO:0004222	metalloendopeptidase activity intron_variant|MODIFIER
F	NKXS01007262	861	0.6732	0.12689	0.1251	-0.1304	**1091.00**	-0.1522	0.0693	0.0198	Haimp10041442m.g	PF00006	ATP synthase alpha/beta family, nucleotide-binding domainsynonymous_variant|LOW
B	NKXS01000006	391845	0.4059	0.018537	**10.0000**	0.1240	2.2507	0.0518	**27.8500**	-0.2410	Haimp10035708m.g	GO:0016760	cellulose synthase (UDP-forming) activityintron_variant|MODIFIER
B	NKXS01001400	41404	0.22740	0.023626	0.1911	0.0867	0.0792	0.0580	**13.9020**	-0.2358	Haimp10009243m.g	-	intron_variant|MODIFIER
B	NKXS01004138	63677	0.13580	-0.072007	0.0908	0.0667	0.1458	0.0983	**46.7970**	-0.1984		-	intergenic_region|MODIFIER|
B	NKXS01000489	3109	0.1340	0.02834	0.0857	0.0877	0.1449	0.0572	**10.3300**	-0.2775	Haimp10030846m.g	PF13432	Tetratricopeptide repeat intron_variant|MODIFIER
B	NKXS01002703	64696	0.1118	0.035165	0.0735	0.0752	0.0528	0.0564	**14.8830**	-0.2750	Haimp10019312m.g	GO:0006777	Mo-molybdopterin cofactor biosynthetic processmissense_variant|MODERATE
A	NKXS01006685	6558	0.0851	0.050983	0.1122	0.0569	0.0775	0.0311	**56.6910**	-0.2467	Haimp10039384m.g	PF00134	Cyclin, N-terminal domain intron_variant|MODIFIER
C	NKXS01003261	39765	0.0825	0.038259	0.0903	0.0468	0.0548	0.0041	**23.1460**	-0.1953	Haimp10022642m.g	GO:0016597	amino acid binding synonymous_variant|LOW
E	NKXS01002889	3697	0.0743	0.0009	0.0772	0.0771	0.0616	0.0558	**18.6630**	-0.1678	Haimp10020435m.g	K15747	cytochrome P450, family 97, subfamily A (beta-ring hydroxylase) ynonymous_variant|LOW
F	NKXS01006483	53149	0.0743	0.0371	0.1034	0.0814	0.0582	0.0379	**13.3330**	-0.2314	Haimp10038613m.g	GO:0005525	GTP binding upstream_gene_variant|MODIFIER
F	NKXS01008537	7108	0.0738	0.0231	0.0539	0.0809	0.0648	0.0344	**34.3730**	-0.2197	Haimp10001901m.g	PF04051	Transport protein particle (TRAPP) componentdownstream_gene_variant|MODIFIER
F	NKXS01003977	34289	0.0716	0.0441	0.0657	0.0222	0.1987	0.0142	**90.3930**	-0.1536	Haimp10026773m.g	GO:0016773	phosphotransferase activity, alcohol group as acceptorintron_variant|MODIFIER
C	NKXS01009331	5768	0.0655	0.0254	0.0572	0.0723	0.0533	0.0643	**35.9380**	-0.2264	Haimp10004318m.g	GO:0005524	ATP-binding missense_variant|MODERATE
C	NKXS01000816	49036	0.0574	0.0355	0.0614	0.0868	0.0524	0.0424	**10.1320**	-0.2472	Haimp10042856m.g	PF01256	Carbohydrate kinase intron_variant|MODIFIER

Scaffold, SNP position, and affected gene were obtained from the genome assembly and annotation (Silva-Junior et al. 2018a, 2018b). Class corresponds to the allele frequency class in Fig. 3. Bio4, temperature seasonality; Bio5, maximum temperature of the warmest month; Bio16, precipitation of the wettest quarter; Bio17, precipitation of driest quarter. SNPEff annotation identifies the SNP position with respect to the genes annotated in the *H. impetiginosus* genome. Upstream and downstream variants are within 5 kb of the transcribed gene regions

**Table 2:** SNPs with potential for adaptive selection based on Bayes factor for soil variables (BF > 10 for at least one soil variable) and correlation (Spearman’s correlation, |*ρ*| > 0.15) using Bayenv2 software

Class	Genbank accession	SNP position	BF CEC Clay	*ρ* CEC Clay	BF CaCO_3_	*ρ* CaCO_3_	BF BS	*ρ* BS	Affected gene	Annotation	Function and SNPEff annotation
A	NKXS01002061	**42170**	**148.7100**	-0.1901	0.3061	-0.0591	1.6204	-0.0417	Haimp10014748m.g	PF00271	Helicase conserved C-terminal domain missense_variant|MODERATE
A	NKXS01001803	**112707**	**47.5550**	-0.1633	0.2172	-0.0440	0.2861	-0.0277	Haimp10012826m.g	GO:0016773	Phosphotransferase activity, alcohol group as acceptor synonymous_variant|LOW|
B	NKXS01001173	**15335**	**11.4750**	-0.1874	0.2047	-0.0198	0.7671	-0.0298	Haimp10006371m.g	GO:0042626	ATPase activity, coupled to transmembrane movement of substances synonymous_variant|LOW|
C	NKXS01005306	29967	2.2584	0.0246	**1.90E** **+** **70**	-0.0757	**3.95E** **+** **19**	-0.1690	Haimp10033515m.g	KOG0032	Ca^2^ + /calmodulin-dependent protein kinase, EF-hand protein superfamily intron_variant|MODIFIER
D	NKXS01001780	62713	0.1414	-0.0079	**2.90E** **+** **53**	-0.1708	**1.17E** **+** **39**	-0.0445	Haimp10012617m.g	KOG1187	Serine/threonine protein kinase synonymous_variant|LOW
D	NKXS01000585	262912	0.1279	0.0096	**39.3780**	-0.1733	3.6111	-0.1007	Haimp10035204m.g	GO:0016616	Oxidoreductase activity, acting on the CH-OH group of donors, NAD or NADP as acceptor intron_variant|MODIFIER
E	NKXS01005306	29965	0.0961	0.0262	**1.26E** **+** **128**	-0.1006	**3.03E** **+** **55**	-0.1861	Haimp10033515m.g	KOG0032	Ca^2^ + /calmodulin-dependent protein kinase, EF-hand protein superfamily intron_variant|MODIFIER
F	NKXS01001446	39931	0.0821	-0.0725	0.5758	-0.1348	**13.7060**	-0.1516	Haimp10009670m.g	KOG2495	NADH dehydrogenase (ubiquinone) downstream_gene_variant|MODIFIER
F	NKXS01002564	31451	0.0806	-0.0802	**16.8870**	0.1048	**84.9890**	0.1741	Haimp10018368m.g	PF01429	Methyl CpG-binding domain intron_variant|MODIFIER
B	NKXS01002428	1817	0.0630	0.0669	**2.21E** **+** **114**	-0.0933	**4.92E** **+** **56**	-0.1661	Haimp10017457m.g	PTHR10996	2-Hydroxyacid dehydrogenase-related intron_variant|MODIFIER|
C	NKXS01002564	35233	0.0542	0.0013	**2.64E** **+** **43**	-0.1935	**5.32E** **+** **18**	-0.1047	Haimp10018369m.g	KOG2253	U1 snRNP complex subunit SNU71 and related PWI-motif proteins downstream_gene_variant|MODIFIER

Scaffold, SNP position, and affected gene were obtained from the genome assembly and annotation (Silva-Junior et al. 2018a). Class corresponds to the allele frequency class in Fig. 3. CEC Clay, subsoil clay cationic exchange capacity; CACO_3_, subsoil clay calcium carbonate; BS, subsoil base saturation. SNPEff annotation identifies the SNP position with respect to the genes annotated in the *H. impetiginosus* genome. Upstream and downstream variants are within 5 kb of the transcribed gene regions

The analysis of selective sweeps and genetic hitchhiking events with SweepFinder2 detected hard selective sweeps within 17 of the 27 genome scaffolds scrutinised, indicating 81 positions along these sequences that may have been targets of selection (Table 3).

**Table 3:** Evidence for selective sweeps based on composite likelihood using the parametric test implemented in SweepFinder2 for the 13 populations of *H. impetiginosus*

Genbank accession	ALTGO	BAGMT	BRAMG	CARMT	MIMGO	MOCMG	NIQGO	PANMG	POFMA	POSGO	POTGO	SECPI	SUMMG	Locus	Distance
NKXS01000006	-	-	-	-	-	112,230 (1.500)	-	-	-	-	-	-	-	Haimp10035675m.g	0
NKXS01000006	-	-	-	-	-	-	-	-	-	419,726 (1.354)	-	-	-	Haimp10035711m.g	0
NKXS01000006	-	-	236,517 (1.427)	-	-	-	-	-	-	-	-	-	-	Haimp10035685m.g	6746
NKXS01000006	-	-	-	-	-	159,053 (7.548)	-	161,198 (1.223)	-	158,525 (1.258)	159,102 (1.666)	-	-	Haimp10035682m.g	0
NKXS01000006	-	-	-	-	-	-	-	-	-	157,428 (1.201)	-	-	-	Haimp10035681m.g	401
NKXS01000006	-	-	260,870 (1.250)	-	-	-	-	-	-	-	-	-	-	Haimp10035691m.g	2005
NKXS01000006	-	-	391,890 (1.840)	-	391,782 (1.884)	-	-	-	-	391,793 (1.595)	-	-	-	Haimp10035708m.g	0
NKXS01000006	-	-	243,743 (1.684)	-	-	-	-	-	-	-	-	-	-	Haimp10035686m.g	0
NKXS01000006	-	-	252,645 (1.421)	-	-	-	-	-	-	-	-	-	-	Haimp10035689m.g	5623
NKXS01000006	-	98,062 (2.260)	-	-	-	-	-	-	-	-	-	-	-	Haimp10035674m.g	0
NKXS01000489	-	32,958 (1.478)	-	-	-	-	-	-	-	-	-	-	-	Haimp10030854m.g	0
NKXS01000489	-	-	-	-	-	-	-	-	-	-	4074 (1.224)	3965 (1.386)	-	Haimp10030846m.g	0
NKXS01000550	-	-	-	-	-	-	-	-	14,596 (1.232)	-	14,740 (1.307)	-	-	Haimp10033596m.g	0
NKXS01000585	-	-	-	-	-	-	-	185,776 (2.026)	-	-	182,515 (2.282)	-	-	Haimp10035192m.g	359
NKXS01000585	-	-	-	-	-	-	-	206,694 (2.031)	-	-	206,696 (2.337)	-	-	Haimp10035197m.g	12,958
NKXS01000585	-	-	-	-	-	-	-	188,681 (2.026)	-	-	188,674 (2.241)	-	-	Haimp10035194m.g	583
NKXS01000585	-	-	-	138,897 (1.239)	-	138,534 (1.403)	-	-	-	-	138,671 (3.325)	138,621 (2.101)	-	Haimp10035183m.g	5312
NKXS01000585	-	198,258 (1.596)	-	-	-	-	-	201,961 (2.032)	-	-	198,209 (3.217)	-	-	Haimp10035195m.g	4501
NKXS01000585	-	-	-	-	107,496 (2.011)	-	-	-	-	-	-	-	-	Haimp10035176m.g	0
NKXS01000585	-	-	-	-	-	-	-	-	121,478 (1.217)	120,102 (1.444)	-	-	-	Haimp10035181m.g	0
NKXS01000585	-	-	-	-	-	-	-	180,136 (1.957)	-	-	-	-	-	Haimp10035191m.g	3828
NKXS01000585	-	-	-	-	-	-	-	186,619 (2.026)	-	-	185,782 (2.258)	-	-	Haimp10035193m.g	0
NKXS01000585	-	-	-	-	-	-	-	172,434 (1.329)	-	-	-	-	-	Haimp10035188m.g	900
NKXS01000585	-	-	-	-	-	-	-	230,503 (1.225)	-	-	-	-	-	Haimp10035198m.g	8583
NKXS01000585	-	-	-	-	-	-	-	174,965 (1.621)	-	-	-	-	-	Haimp10035190m.g	449
NKXS01000816	-	-	-	-	-	-	-	48,479 (1.702)	-	-	49,419 (2.354)	49,055 (1.915)	-	Haimp10042856m.g	0
NKXS01001173	15,471 (1.698)	-	15,646 (1.788)	-	-	-	-	-	15,159 (1.441)	-	15,647 (1.233)	15,412 (2.193)	-	Haimp10006371m.g	0
NKXS01001173	-	-	-	-	-	-	-	-	88,292 (1.441)	-	-	-	88,217 (2.107)	Haimp10006385m.g	0
NKXS01001173	-	-	-	-	-	111,029 (2.256)	-	-	-	-	111,073 (1.202)	-	-	Haimp10006388m.g	0
NKXS01001400	-	-	-	-	-	-	-	-	-	-	41,407 (1.644)	-	-	Haimp10009243m.g	0
NKXS01001400	-	-	-	-	-	-	-	-	-	-	23,526 (1.621)	-	-	Haimp10009240m.g	0
NKXS01001400	-	57,194 (1.459)	-	-	-	-	-	-	-	-	-	-	-	Haimp10009245m.g	165
NKXS01001803	50,581 (1.349)	50,500 (1.489)	-	-	-	-	-	50,608 (1.422)	-	-	-	-	-	Haimp10012819m.g	0
NKXS01001803	-	-	-	-	-	-	-	-	112,936 (1.413)	-	112,016 (1.281)	-	-	Haimp10012826m.g	0
NKXS01002428	-	-	-	-	-	-	1,553 (1.703)	-	-	-	-	-	-	Haimp10017457m.g	0
NKXS01002428	-	-	-	-	-	27,811 (1.591)	-	-	-	-	-	-	-	Haimp10017464m.g	0
NKXS01002564	-	31,699 (2.360)	-	-	-	25,828 (2.613)	-	-	-	-	25,200 (5.889)	-	-	Haimp10018368m.g	0
NKXS01003977	-	55,907 (1.463)	-	-	-	-	-	-	-	-	-	-	-	Haimp10026776m.g	0
NKXS01003977	-	-	-	-	-	-	-	-	-	-	41,373 (1.975)	41,703 (1.397)	-	Haimp10026773m.g	0
NKXS01005306	-	-	2672 (1.517)	-	-	-	-	-	-	-	-	-	-	Haimp10033514m.g	0
NKXS01006483	-	-	-	-	-	-	-	-	-	-	50,677 (1.295)	-	-	Haimp10038613m.g	0
NKXS01006483	-	-	53,115 (1.217)	-	-	-	-	-	-	-	-	-	-	Haimp10038614m.g	0
NKXS01006685	-	-	-	-	-	-	-	-	-	-	6569 (1.602)	-	-	Haimp10039384m.g	0
NKXS01008263	-	-	-	-	-	-	-	-	-	-	19,942 (1.544)	-	-	Haimp10001090m.g	0
NKXS01008537	-	-	-	-	-	-	-	-	-	-	11,614 (1.246)	-	-	Haimp10001901m.g	0
NKXS01008537	-	-	-	-	-	-	-	-	-	-	7125 (3.171)	-	-	Haimp10001900m.g	0
NKXS01009331	-	-	-	5188 (1.551)	5622 (1.705)	-	-	5104 (1.265)	-	-	-	-	5801 (1.454)	Haimp10004318m.g	0
Number of loci	2	7	8	2	3	6	1	13	5	5	22	5	2	47	

The values are the location and the calculated composite likelihood. Only the most extreme signals above the significance cut-off are summarised in terms of the most probable location of the sweep to the nearest gene locus annotated in the genome assembly of the species. Distance is the minimum distance between the location of the sweeps in the populations and the closest locus. Zero (0) values mean that the location of the most extreme signal is within the gene locus

### Functional annotation of SNPs with selection signal

Among the 14 outlier SNPs identified by Outflank for cluster C2, only one does not affect the transcribed region (Table 4) of a gene. According to the SNPEff annotation, all the other 13 SNPs (~93%) potentially affect the transcribed region (UTR, exons or introns) of 11 different genes. Five of these genes (46%) have SNP in introns, two (18%) in the UTR (5’- or 3’-untranslated) regions, and four (36%) in exons or coding sequences. Two of the coding sequence SNPs are non-synonymous, one generates a gain of stop codon, and another is non-synonymous.

**Table 4:** SNPs with potential for adaptive selection based on the analysis of five populations from structure cluster C2, using Outflank software

Genbank accession	SNP position	Affected gene	Annotation	Function and SNPEff annotation
NKXS01001300.1	46,609	Haimp10008207m.g	GO:0000162	Tryptophan biosynthetic process/LOW
NKXS01012733.1	756	Haimp10011772m.g	GO:0010542	Nitrate efflux transmembrane transporter activity/MODIFIER
NKXS01002024.1	84,206	Haimp10014472m.g	GO:0008270	Zinc ion binding/MODERATE
NKXS01002456.1	149,948	Haimp10017658m.g	GO:0000301	Retrograde transport, vesicle recycling within Golgi/LOW
NKXS01002755.1	29,846	Haimp10019594m.g	GO:0031625	Ubiquitin protein ligase binding/MODERATE
NKXS01003907.1	15,012	Haimp10026309m.g	GO:0006970	Response to osmotic stress/LOW
NKXS01000398.1	82,059	Haimp10026508m.g	GO:0000166	Nucleotide binding/LOW
NKXS01004502.1	42,878	Haimp10029651m.g	GO:0046872	Metal ion binding/LOW
NKXS01004933.1	37,022	Haimp10031868m.g	GO:0007264	Small GTPase-mediated signal transduction/LOW
NKXS01005590.1	650	Haimp10034961m.g	GO:0001085	RNA polymerase II transcription factor binding/MODIFIER
NKXS01006140.1	23,081	Haimp10037322m.g	GO:0034314	Arp2/3 complex-mediated actin nucleation/LOW

Scaffold, SNP position, and affected gene were obtained from the genome assembly and annotation (Silva-Junior et al. 2018a, 2018b). SNPEff annotation identifies the SNP position with respect to the genes annotated in the *H. impetiginosus* genome. Upstream and downstream variants are within 5 kb of the transcribed gene regions

GO annotation of these 11 genes pointed to a wide spectrum of biological processes. These include the response to osmotic stress, acid pH, and organic substances, and nitrate transport, protein deubiquitination, regulation of transcription, Golgi-mediated transport, pollen-tube growth, phenylalanine-tRNA ligase activity, and actin nucleation (Table 4).

Bayenv2 detected 32 SNPs via their correlation with environmental variables. Among the 32 SNPs, 21 were correlated with at least one of the selected climate variables, and 11 with soil variables. SNPEff annotation of these SNPs indicated that 27 genes are potentially affected by these variations at the DNA level.

The 21 loci correlated with climate variables were found within or in close proximity (within 5kb upstream or downstream of the transcribed region) to 17 genes. Most genes (14 of 17; 82%) had at least one SNP within their transcribed regions (exons and introns; Table 1), while three genes (18%) had the nearby SNPs outside the transcribed region. One gene, Haimp10041442m.g (Table 1), had SNPs located within the transcribed region and in close proximity to the transcription start site. Considering the SNPs located within the genes (15 of 21 or 71%), SNPEff indicated that seven were within the exons, with two inducing missense and five synonymous mutations. The remaining eight SNPs were all found in the introns (Table 1).

The 11 SNPs correlated with soil variables potentially affect 10 genes (Table 2). Eight of these genes (80%) contain SNPs within exons or introns, while the remaining two genes (20%) have SNPs within 5kb upstream or downstream of the transcribed region. Of the four SNPs found within coding regions, one induces missense mutation and three synonymous mutations.

GO annotation of the 27 genes potentially affected by the 32 SNPs with putative signals of adaptive selection revealed their involvement in a wide spectrum of underlying biological processes (Supplementary File S2 and Tables S3 and S4). These include housekeeping genes such as adenosine 5’-triphosphate (ATP) synthase, tRNA synthetase, and helicase, as well as the genes involved in mitotic checkpoint, microbule movement (kinesin), and ATPase activity. There were also genes involved in transcription regulation (CCAAT-binding factor), cell-wall formation (cellulose synthase), signal transduction (several kinases), as well as oxidation–reduction process (cytochrome P450, NADH dehydrogenase, and glyoxylate/hydroxypyruvate reductase). Interestingly, a Ca^2 + ^/calmodulin-dependent protein kinase, Haimp10033515m.g (Table 2), had two SNPs, correlated with soil variables, within its introns.

In the SweepFinder2 analysis, 70% of the maximum likelihood estimates of the sweeps fell inside 33 protein-coding genes while 30% were found within 12.9kb upstream or downstream of the transcribed region of the other 14 gene loci. Thus, a total of 47 genes have the putative loci under selection (Table 3). Fifteen of these genes are common to the Bayenv2 analysis. GO enrichment analysis using tests based on the hypergeometric distribution (*p* < 0.1) in the FUNC program (Prüfer et al. 2007) shows prominent roles of genes, with GO terms related to complex cellular functions involving large networks of interactions, such as ribosome assembly (UtpB chaperone), transcription factors (CCAAT box-binding factor), post-translational protein modification (ubiquitin-activating enzyme complex), regulation of enzyme activity (B56 regulatory subunit of protein phosphatase 2A), and degradation of intracellular proteins (20S central proteasome complex). Other noteworthy terms include enzymes in the biosynthetic pathways of specialised metabolites such as terpenoid and steroid (Supplementary File S2 and Table S5).

### Spatial prediction and shifts in allele frequency

**Fig. 2:**
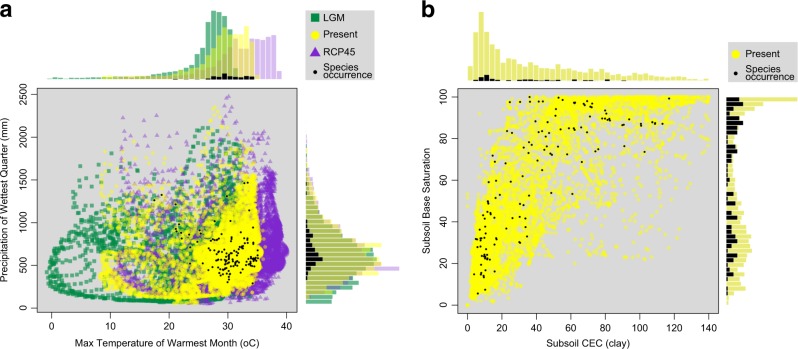
Ecological space of climatic and soil conditions in the Neotropics for *H. impetiginosus*. **a** Climatic niche space during the LGM (21ka, green squares), present day (yellow dots), and the EOC (2100, purple triangles). The climatic preferences from the current occurrence records of *H. impetiginosus* are represented by black dots. The climatic conditions matching the species preferences were less available during the LGM than the present day, mainly due to temperature decrease. **b** Soil niche space for the present day (yellow dots) and current occurrence (black dots). CEC is the subsoil clay cationic exchange capacity. Note the high variation in soil conditions matching the species preferences. Occurrence records of *H. impetiginosus* were obtained from the online databases GBIF (Global Biodiversity Information Facility, http://www.gbif.org/). EOC, end of century; LGM, last glacial maximum

The present-day climatic conditions across the geographical range of *H. impetiginosus* show its preference for hot and drier climates, matching the current general conditions of SDTFs (Fig. 2a). Such a climatic space was slightly different during the LGM due to a temperature decrease, and is expected to be different at the EOC due to an increase in temperature and decrease in precipitation (Fig. 2a; Supplementary File S3 and Fig. S5). However, *H. impetiginous* shows no obvious preference for soil fertility (Fig. 2b; Supplementary File S3 and Fig. S6), occurring in a wide range of soil CEC and BS.

**Fig. 3:**
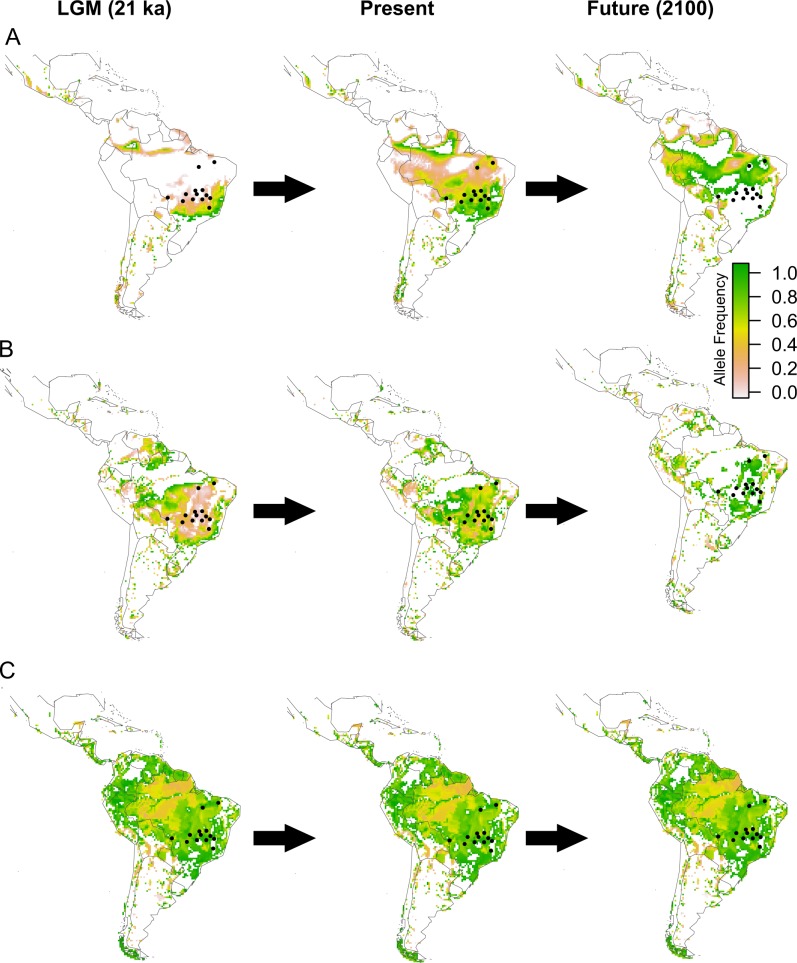

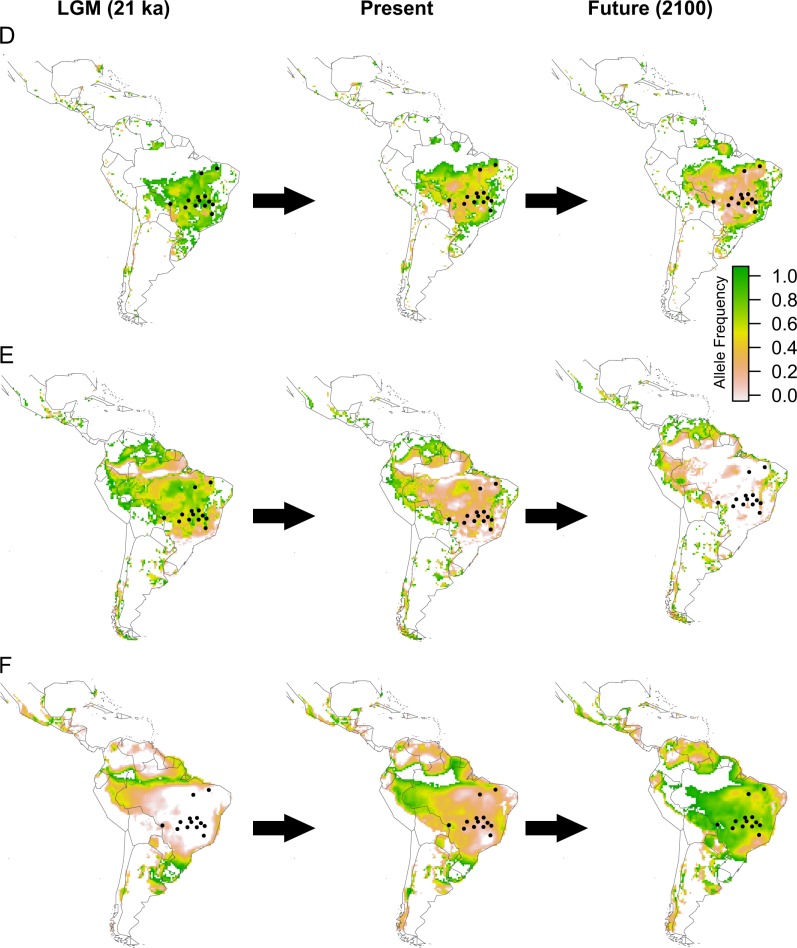
Expected allele frequency for loci under natural selection based on spatial interpolation for environmental condition at the last glacial maximum (LGM), the present day, and the end of century (2100, EOC) using RCP 4.5. Shown are the spatial patterns for six classes of loci (A–F), representing the behaviour of the 32 loci with higher correlation with environmental variables. See Tables 3 and 4 for loci in each class

Predicted values of allele frequency using spatial interpolation for the 32 SNPs having correlation with environmental variables show changes in allele frequency through space and time. Co-kriging to the Neotropics analysis based on the pattern of spatial distribution of allele frequency through time allowed us to classify the 32 SNPs into six categories according to the similar spatial pattern of allele frequency distribution, depicted as class A to F in the maps (Fig. 3). SNPs within each class of allele frequency correlated to the environmental (Table 1) and soil (Table 2) variables are listed.

**Fig. 4:**
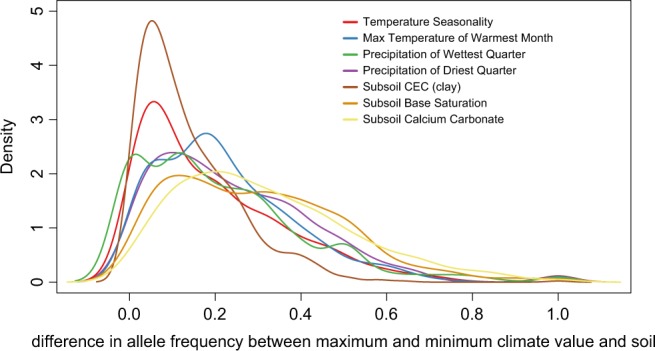
Comparison of the distributions of shifts in allele frequency for the 32 SNPs with selection signatures with the highest correlation with environmental variables. The shift is defined as the difference between the allele frequencies of the populations with the highest and the lowest value for each climatic and soil variable. The density function was obtained for the vector of differences in allele frequency for the loci and calculated using the function *density* implemented in the *stat* package in R 3.6.0

Maps of the present-day expected frequency show a tendency of the fixation of one allele across the studied populations for many loci, such as the loci in class C, E, and F (Fig. 3). The expected frequency of alleles is spatially structured for some loci (loci in class A and D; Fig. 3), with difference in allele frequency between the SDTFs from Central Brazil and east Amazonia and Atlantic forest. Most loci under selection showed only a slight difference in allele frequency between populations with different climatic and soil conditions (Fig. 4). In the maps of the LGM and EOC, most loci showed changes in the predicted allele frequency through time (Fig. 3). Loci from class A and E show sharp changes in allele frequency through time and unsuitable conditions at EOC whereas loci from class F show unsuitable conditions at the LGM and a highly different allele frequency at present time and EOC.

## Discussion

We generated genome scan data across 13 populations of *H. impetiginosus* covering a wide geographical range using 75,838 high-quality SNPs. The data were analysed under three different approaches in an attempt to detect the loci under selection. The Outflank approach detected 14 outlier loci for adaptive selection in a specific cluster of populations displaying lower genetic differentiation. Correlations with soil and climate variables provided evidences for 32 loci underlying local adaptation, although correlations of their allele frequencies with bioclimatic variables were low. Using SweepFinder2 to test the hypothesis of recent selective sweeps along genome scaffolds targeted by SNPs, we were able to provide additional mapping of putative adaptive changes in the genome of *H. impetiginosus*. Consistent with the Bayenv2 analysis, few loci were detected indicating positive selection, considering the number of loci genotyped and the transcriptome size (nearly 32k genes; Silva-Junior et al. 2018a) of *H. impetiginosus*. Additionally, the evidence for selective sweeps based on the composite likelihood calculation was not strong as the highest values surpassed the significance cut-off by only twofold. Moreover, many of the genes potentially affected by the SNPs detected are related to highly conserved primary metabolic processes or housekeeping functions, the underlying genes of which are likely under strong purifying selection (Siol et al. 2010). It should be noted that SweepFinder2 is specifically designed to detect the loci undergoing selective sweeps and may have little power to detect other types of selection (Nielsen et al. 2005). These results, taken together with the significant genetic differentiation observed among populations, the demographical and range expansion during glaciations in the Quaternary and small effective population size (Collevatti et al. 2012), led us to propose that other types of soft selective forces such as background, balancing, and purifying selection are probably occurring. However, they are hard to detect given the limited power of our experimental design and the currently available analytical tools.

### Evidences of selection sweeps for adaptation were largely detected in highly diverse but structurally conserved genes

Many of the genes nearest to the location of the sweeps have fundamental roles in cells and are observed to be highly conserved during evolution, such as the proteasome and ubiquitin-activation complexes, the modular transcriptional activator HAP complex (Nuclear Factor Y, subunit HAP5), and members of the ATP-binding cassette (ABC) superfamily of transporters. Although highly conserved during evolution, these complexes are large connected systems that are thought to have diversified into functional distinctive groups. Examples in vertebrates include the proteasome system in adaptive immunity (Tanaka, 2009), and members of ABC superfamily in humans (Wang et al. 2007), other mammals, and fishes (Fischer et al. 2013). In plants, multiple proteasome core genes, transcription factor genes, and classes of transmembrane protein-coding genes such as the ABC superfamily are found retained after events such as diploidization, and local or segmental duplication, though their biological roles are still under scrutiny. The ABC transporter gene found in our analysis of recent selective sweeps, for instance, is located in a region harbouring another gene locus potentially coding for a protein member of this superfamily, which suggests that they may have arisen through gene duplication. Plant CCAAT box-binding factors seem to have diversified into at least two main groups (Laloum et al. 2013), and their subunits are encoded by multigene families, the members of which show structural and functional diversifications with implication to developmental processes and response to environment (Zanetti et al. 2017). Evolutionary models such as the gene balance hypothesis, which aims to predict the selective consequences of duplication events, indicate that these connected genes encoding the subunits of a complex are particularly dosage-sensitive. Consequently, there should be selection for any successful adaptation that mitigates the gene-content changes following duplication (Freeling and Thomas, 2006). Regulatory genes, the major fraction of the loci in our enrichment analysis, constitute a standard case falling into this general concept (Birchler and Veitia, 2010).

Interestingly, some proteins highlighted in our analysis of selection footprints are enzymes with catalytic activity, which is a process that requires metabolic energy. ATP and other carrier molecules such as NAD(P)H play a central role in this process, rapidly diffusing throughout the cell to the sites where energy is used for catalytic activities (Alberts et al. 2002). Bayenv2 and SweepFinder analyses, for instance, pointed to putative adaptive SNPs within the genes, the products of which are molecules directly involved in the production of ATP (ATP synthase and ubiquinone). It is also important to note that the genome of *H. impetiginosus* was found to be enriched for genes involved in metabolic processes and catalytic activity in comparison with other species of the Lamiids, such as *Erythranthe guttata* and *Olea europaea* (Silva-Junior et al. 2018a), which suggests the potential response of enzymatic activity to environment variables (Sulpice et al. 2010). Calcium/calmodulin-dependent protein kinases, for instance, are a diverse but structurally conserved family of enzymes that constitute the plant Ca^2+^-signalling toolkit. Ca^2+^ is known to act as a second messenger in cellular signalling networks underlying processes that make plants complete their life cycle, reproduce, and respond to environmental factors like geotropism and growth towards light, water, and nutrients from the soil (Edel et al. 2017).

Given the clustered nature of the sequence-capture approach, most pair-wise SNPs of a given scaffold are within close distances. Even though linkage disequilibrium (LD) when measured at short distances tends to be underestimated due to gene conversion (Andolfatto and Nordborg, 1998), *H. impetiginosus* does show a very steep decline of LD (Supplementary File S3 and Fig. S5). This low LD is consistent with its outcrossing nature and high genetic diversity (Collevatti et al. 2012), and should contribute to increasing the resolution of population genomics studies. We found four SNPs with the adaptive selection signal mapped to the same gene (Haimp10041442m.g; Table 1), which is most likely due to the sampling SNP density, along with the timing and strength of the sweep that captured these SNPs.

### *H. impetiginosus* may track future environmental changes

A subset of 200 putatively neutral SNPs recovered the high differentiation among populations corroborating the previous results of high genetic differentiation (Collevatti et al. 2012) using both presumed neutral nuclear ribosomal DNA (*F*_ST_ = 0.808) and chloroplast (*F*_ST_ = 0.891) intergenic spacer sequences. Bayesian clustering of populations based on neutral SNPs showed an east to west differentiation in allele frequency (Fig. 1) with a high admixture. Additionally, our simulations using neutral SNPs recovered the same demographical history as previously shown by phylogeographical analyses (Collevatti et al. 2012), i.e. a wide demographical expansion in the LGM followed by a retraction leading to the current disjunction distribution and small effective population sizes (Collevatti et al. 2012). This expansion may have led to the widespread distribution of alleles and may explain the sharing of alleles among populations, even under different climatic and soil conditions (Supplementary File S1 and Table S7).

Contrasting environments for bioclimatic variables showed little difference in allele-frequency distribution in *H. impetiginosus*. Only slight differences in allele frequency were found among the sampled populations in SDTFs from different regions, such as Central Brazil (e.g. populations ALT, BAG, and CAC), Eastern Amazonia (population POF), and the eastern most region of Brazil along the Atlantic forest (SEC), despite the environmental differences. On the other hand, a large difference in environmental conditions over time is expected to have occurred for *H. impetiginosus*, from the LGM to the present day, potentially changing the patterns of the expected allele frequencies, as forecasted by our simulations. We acknowledge, however, that our simulation is a simplification of the evolutionary process, because of the arbitrary assumptions that selection is constant through time, and only environment variables change.

The small differences in allele frequency distributions among contrasting environments for many loci suggest the potential ability of the species to cope with the impact of climate changes. It is also reasonable to suggest that the species will potentially be able to track environmental changes or will be largely unaffected due to its phenotypic plasticity (Agrawal, 2001). The small shifts among populations observed in allele frequency distribution may also be taken as an indication of soft selective sweeps for many loci. In geographically structured populations, several mutations can emerge independently in different populations or the standing variation may be sorted among populations, and soft sweeps are possible due to parallel adaptations in which multiple mutations may lead to similar phenotypes (Arendt and Reznick, 2008), which may involve the sweep locations inferred from our data (Table 3). As a consequence, diversity is not necessarily reduced, and the difference in allele frequencies among populations may be low compared to hard selective sweeps (Messer and Petrov, 2013). Moreover, demographical dynamics due to environmental changes over time may change the adaptive landscape, and neutral or deleterious alleles may become beneficial in different populations (Przeworski et al. 2005). Canonical genome-scan approaches are typically based on the assumption of positive selection on a mutation leading to hard selective sweeps with limited power to detect other kinds of selections such as soft sweeps (Messer and Petrov, 2013).

Our results are somewhat in contrast with those reported in population genomics studies of forest tree species in temperate regions. Typically, distinct adaptive signals can be detected for photoperiod-responsive and dormancy- or temperature-related traits that show strong latitudinal clines and are critical for species survival (e.g., Yeaman et al. 2014; Parchman et al. 2012; Hornoy et al. 2015). The tropical environment, on the other hand, is strongly associated with the notion of physical and chemical stability (Barron, 1995) and lack of latitudinal clines such that hard selective sweeps are more unlikely to occur and soft sweeps are to be detected.

Unfortunately, at this point, there are no other landscape genomics studies on tropical trees to help support this proposition, which underscores the contribution of our study and reinforces the necessity of more investigations in tropical biomes. However, we can at least speculate that *H. impetiginosus*, as a representative widespread species in the tropics, may be responding to the tropical environmental variation more by polygenic adaptation and phenotypic plasticity. Polygenic adaptation would allow effective adaptation to environmental changes precluding selective sweeps and with little effect on substitution rates (Pritchard and DiRienzo, 2010). The detection of genomic signals of polygenic adaptation is currently a hot but still experimentally challenging theme in evolutionary genetics, likely to face major advances in the coming years (Csilléry et al. 2018). Theoretical and empirical evidence has emerged, which suggests that modularity in developmental genetic networks should underlie phenotypic plasticity. Modular biological organisation, whether at the level of genes or at the level of traits, is predicted to evolve, at least in part, in response to environmental variation (Snell-Rood et al. 2010).

Our spatial interpolation (co-kriging) analysis of the expected allele frequency across the SDTFs indicated that changes in environmental landscape in the future might impose adaptive challenges to *H. impetiginosus*. However, the species showed potential for range shifts over time. Our findings show that, for many loci, suitability will potentially decrease sharply in EOC in some parts of the geographical range of *H. impetiginosus*. For instance, the environmental space will change at the EOC and will not match the present and past conditions that favoured heterozygous or alternative alleles for some loci (e.g. loci matching the patterns of class A and E; Fig. 3) in most geographical range. The lack of suitable environmental conditions could therefore challenge the permanence of *H. impetiginosus*, hindering the response to selection in a changing environment. However, not only the dependence of the species on range shifts towards more suitable conditions in other Neotropical environments (Collevatti et al. 2012), but also phenotypic plasticity, not taken into account in our modelling of response to climate change, might play a key role in enabling the persistence of remnant individuals or populations.

In conclusion, what we have carried out, to the best of our knowledge, is the first genome-wide population genomics study of a Neotropical tree species. Our results point to the likelihood of local adaptation at a few specific loci that were within the detectable level of our sample size and confounding genetic structure background. Being aware of the limits of our experiment, it is also worth noting that other softer selective forces such as background, balancing and purifying selection, largely undetectable, cannot be dismissed at this point, as the key components in shaping local adaptation in *H. impetiginosus*. These results, in turn, are consistent with the concept that adaptive traits are largely under polygenic control in natural populations (Pritchard and DiRienzo, 2010) involving many loci, the vast majority with small effects, and are likely subject to stabilising selection towards an intermediate optimum as substantiated by classical analyses (Fisher, 1930; Wright, 1935). Finally, this study leveraged the power of sequence-capture SNP genotyping together with the availability of a well-curated whole-genome sequence assembly to generate data for over 75,000 SNPs identified within or in close proximity to nearly 7,000 protein-coding genes predicted in the genome assembly of a species. This approach has now become more accessible and should therefore be widely applicable to unravel the evolutionary history of several other tropical forest tree species, adding much needed knowledge towards a deeper understanding of the megadiverse forest biomes.

### Data archiving

Additional data are provided as supporting information in the online version of this article.

## Supplementary information

Supplemental File S1

Supporting Information S1 - Tables

Supporting Information S2 - Tables

Supporting Information S3 Figures
