# Supplementary material for: A genome-wide scan shows evidence for local adaptation in a widespread keystone Neotropical forest tree
Source: Heredity (Edinb). 2019 Feb 12;123(2):117–37. doi: 10.1038/s41437-019-0188-0 (PMC6781148; doi:10.1038/s41437-019-0188-0)
Supplement: Supplementary file 1 — Supplemental File S1 [file 41437_2019_188_MOESM1_ESM.docx]

**Supplementary File S1 for**

**A genome-wide scan shows evidence for local adaptation in a widespread keystone Neotropical forest tree**

Rosane G. Collevatti, Evandro Novaes, Orzenil Bonfim da Silva-Junior, Lucas Vieira, Matheus S. Lima-Ribeiro, Dario Grattapaglia

**Sequence capture and SNP genotyping pipeline**

A detailed description of the sequence capture and SNP genotyping pipeline and quality control can be found in (Silva-Junior et al., 2018) and a brief summary is presented here.

Targeted DNA-enrichment, capture and sequencing were carried out by RAPiD Genomics LLC (Gainesville, FL) using the SureSelectXT (Agilent Technologies, CA) enrichment system followed by Illumina sequencing as described earlier (Neves et al., 2013). Briefly, genomic DNA libraries were prepared using the Agilent protocols, including DNA shearing followed by ends repair, 3’-end adenylation, adaptor ligation, and amplification. Agencourt AMPure XP beads were used to purify the libraries following each step. Library was enriched by hybridization to the designed capture probes according to the Agilent protocol, with custom modification of concentration, adapter sequence. Custom barcode adapters were used to pool up to eight samples in a single hybridization. Following hybridization, target regions were purified with magnetic beads and amplified for enrichment and addition of index sequences. Libraries were pooled in equimolar amounts and sequenced in 2 and 2/3 lanes of an Illumina HiSeq2000 instrument (Illumina, CA), 1 x 101 bp mode. Sample multiplexing for sequencing was achieved using 48 individual barcode sequences per lane, one for each sample from 128 individuals of Handroanthus impetiginosus.

Sequencing reads were trimmed for adapters using fastq-mcf in the ea-utils package (Aronesty, 2013). Adapter-free reads were aligned to all the scaffolds in the genome sequence assembly of H. impetiginosus (Silva-Junior et al., 2018a) with BWA version 0.5.9 (Li & Durbin, 2009) using BWA-backtrack algorithm, default settings. These steps were performed in a grid computing cluster software system using a pipeline available from the International Cassava Genetic Map Consortium (ICGM, 2015). Joint-sample genotyping was performed using GATK-GenotypeGVCFs (McKenna et al., 2010) method on gVCF files produced by GATK-HaplotypeCaller (arguments: -mbq 10 -mmq 10 -hets 0.018 -ERC GVCF) in the GATK suite of programs version 3.7-0. For the arguments, mbq is the minimum base quality required to consider a base for calling, default 10 (https://software.broadinstitute.org/gatk/documentation/tooldocs/3.7-0/org_broadinstitute_gatk_tools_walkers_haplotypecaller_HaplotypeCaller.php#--min_base_quality_score); mmq is the minimum read mapping quality required to consider a read for calling, default 10 (<https://software.broadinstitute.org/gatk/documentation/tooldocs/3.7-0/org_broadinstitute_gatk_engine_filters_MappingQualityFilter.php>). The parameter hets in GATK is analogous to the parameter theta, the population scaled mutation rate, from population genetics (https://software.broadinstitute.org/gatk/documentation/article?id=11035). We used mlRho program (Haubold et al., 2010) to obtain estimates of theta from the sequence data on the sampled populations and the entire procedure is described elsewhere (Silva-Junior et al., 2018b). The resulting VCF file, containing SNP calls for all H. impetiginosus samples was tagged to identify unreliable data using a combination of filters. First, variant quality recalibration (VQSR) for detected SNPs was carried out to filter sites likely to be data processing artefact instead true genetic variant. Following the GATK Best Practices recommendations (DePristo et al., 2011), this procedure used a database of true sites provided as input, which was found to be polymorphic on the evaluation of the capture system in a partially replicated genotyping assay using high sequencing coverage (>70x) across target loci in a representative sample of 24 individuals from six populations of H. impetiginosus (Silva-Junior et al., 2018b). After variant score recalibration, genotypes of samples at each SNPs passing VQSR filtering were set to null if genotyping quality was less than 20 (minGQ = 20). Only polymorphic sites and maximum of 20% of missing genotype data were kept.

**Details of Structure analysis**

We also accessed the genetic structure of the populations using the 200 putatively neutral loci to obtain the most likely number of genetic clusters (K) using Structure 2.3.4 (Pritchard et al., 2000). Five independent runs for each K (1–13) were performed to evaluate the consistency of the results using the admixture model of ancestry and correlated allele frequencies. The simulations were performed with a burn-in of 100,000 replications, to minimize the effect of the random starting configuration, followed by 1 million Markov chain Monte Carlo (MCMC) iterations for data collection. To assess the most likely number of clusters supported by the data, we used ∆K following Evanno et al. (2005).

**Parameters of demographical history simulation**

We modelled four demographical scenarios following the framework described in Collevatti *et al*. (2012, 2013). i) the `Pleistocene Arc Hypothesis` (Prado and Gibbs, 1993), an expansion throughout the Central and Southwest Brazil; ii) the `Amazon SDTF Hypothesis` (Pennington *et al*., 2000), a westward range shift, toward the Amazon Basin; iii) Both expansions (‘Both’ hereafter), i.e. a prediction for the past distribution as expected by Pleistocene Arc and Amazon SDTF hypotheses, resulting in an expansion throughout the Central and Southwest Brazil and also towards the interior of Amazon Basin (see Collevatti *et al*., 2012, 2013); and iv) “Range Retraction”, a hypothesis derived from ecological niche modelling published elsewhere (Collevatti *et al*., 2012), i.e. a retraction in geographical range in Central Brazil but without range shift.

For each demographical scenario, we ran 2,000 independent simulations for 200 putatively neutral SNPs. The number of generations until the LGM (Last Glacial Maximum, at 21 ka) was calculated using a generation time of 15 years (Collevatti *et al*., 2012). Demographical hypotheses were simulated backward, with 13 demes from time t0 (present) to t1400 generations ago (at the LGM) using the same parameterization described in Collevatti *et al*. (2012). Effective population sizes at time t (Nt) were calculated from Nt = {ln(N1/N0)/[t]}, where N0 = 10,000 (effective population size at present) was the same for all scenarios, and N1400 (effective population size 1400 generations ago, at the LGM) varied among the hypotheses according to the theoretical expectation. For "Both" and Pleistocene Arc hypotheses we simulated a demographical expansion with N1400 = 50,000 and N1400 = 10,000, respectively. For Amazon SDTF hypothesis we simulated a retraction with extinction of some demes with N1400 = 1,000 and for "Range Retraction" we simulated a stronger retraction with extinction of all demes except one, with N1400 = 1,000. To simulate migration we considered a finite island model in which all current demes are descendants from lineages originally in deme 1 at t generations ago, meaning that as the tree builds back through time, there is a 0.01/generation chance that each lineage in deme x will migrate to deme 1.

Simulated alternative models were compared based on the distribution of expected heterozygosity in the 2,000 simulations. We estimated two-tailed probabilities as twice the number of diversity estimates that were higher than the observed, divided by the number of simulations, so that a high p value indicates failure to reject the model. We also estimated the Akaike information criterion (*AIC*) for model choice. The log-likelihood, ln(L), was estimated as the product of the height of the empirical frequency distribution at the observed value of diversity by the maximum height of the distribution. *AIC* (-2Ln(L) + 2K, where K is the number of free parameters (2 for all models), was transformed into *AIC* weight of evidence (*AICw*), by exp[-0.5(AIC – AICmin)] (Burnham and Anderson, 2002). We also obtained *ΔAIC*; i.e. the difference of *AICw* between each model and the best model. Models with *ΔAIC*< 2 were considered as equally plausible to explain the observed pattern (Zurr *et al*., 2009) and *AICw* was expressed as a relative value among models.

**Analytical procedure and parameters used for SweepFinder2**

We considered data without structure, that is, where the SNP data have been obtained from each single population of the 13 sampled sites. We assumed unknown polarity for any SNP and considered that the minor allele is the derived allele for a particular SNP. For each scaffold, the CLR function was calculated on a grid of 10,000 positions along the length of the sequence. The CLR test statistic used to test for selective sweep was the maximum composite likelihood value optimized over possible positions, compared to the composite likelihood of the neutral null model as calculated by the program. To gain information on critical values of the composite likelihood of the neutral model we simulated neutral new data sets with values of the population scaled mutation rate (θ) and population scaled recombination rate (ρ) typical for highly heterozygous tropical forest trees estimated from genome-wide data (Silva-Junior and Grattapaglia, 2015). We used $\theta/{bp}=0.018 {bp}^{-1}$ and $\rho/{bp=0.0011 {bp}^{-1}}$, assuming homogenous rates among regions. Samples with the same number of individuals in the real populations and sequence length of scaffolds were generated under neutrality using ms (Hudson, 2002) and Seq-Gen (Rambaut and Grassly, 1997) with 100 replicates per scaffold. For each replicate, multiple alignment was performed using MAFFT v7.130b (Katoh and Standley, 2013) and variant sites were detected across the samples using SNP-sites (Page *et al*., 2016). The entire inference procedure to obtain composite likelihoods was repeated for each replicate with SweepFinder2 using the same grid size as for the real data. Composite likelihood values were collected from all the replicates across all positions along the length of the simulated sequences totalling 27 million of data points. A cumulative histogram of the values was used to count the cumulative cases and ascertain what is the critical composite likelihood value for which 95% of the cumulative cases on neutral data lies within. The value of 1.40 was used as the significance cut-off. The probable locations of sweeps were taken as the maximum composite values above the significance cut-off calculated for each scaffold. Positions across sequences and populations were summarized according the distance of the sweeps to the closest gene model in the genome assembly using *bedtools* program (Quinlan and Hall, 2010).

**References**

Aronesty, E. (2013). Comparison of sequencing utility programs. The Open Bioinformatics Journal 7 (1), 1-8.

Burnham KP, Anderson DR (2002). Model selection and multimodel inference: a practical information-theoretic approach. Springer, New York.

Collevatti RG, Terribile LC, Lima-Ribeiro MS, Nabout JC, Oliveira G, Rangel TF, Rabelo SG, Diniz JAF (2012). A coupled phylogeographical and species distribution modelling approach recovers the demographical history of a Neotropical seasonally dry forest tree species. *Molecular Ecology* **21**: 5845-5863.

Collevatti RG, Terribile LC, Lima-Ribeiro MS, Nabout JC, Rangel TF, Diniz-Filho JAF (2013). Drawbacks to palaeodistribution modelling: the case of South American seasonally dry forests. *Journal of Biogeography* **40**: 345-358.

DePristo M, Banks E, Poplin R, Garimella K, Maguire J, Hartl C, [Philippakis](https://www.nature.com/articles/ng.806#auth-7) AA, [del Angel](https://www.nature.com/articles/ng.806#auth-8) G,  [Rivas](https://www.nature.com/articles/ng.806#auth-9) MA, [Hanna](https://www.nature.com/articles/ng.806#auth-10) M, et al. (2011). A framework for variation discovery and genotyping using next-generation DNA sequencing data. *Nature Genetics* **43**: 491-498.

Evanno G, Regnaut S, Goudet J (2005). Detecting the number of clusters of individuals using the software STRUCTURE: A simulation study. *Molecular Ecology* **14**: 2611–2620.

Haubold B, Pfaffelhuber P, Lynch M. (2010). mlRho - a program for estimating the population mutation and recombination rates from shotgun-sequenced diploid genomes. Mol Ecol, 19 Suppl 1, 277-284.

Hudson RR (2002). Generating samples under a Wright-Fisher neutral model of genetic variation. *Bioinformatics* **18**: 337-338.

ICGMC 2015, High-Resolution Linkage Map and Chromosome-Scale Genome Assembly for Cassava (*Manihot esculenta* Crantz) from 10 Populations. *G3: Genes|Genomes|Genetics*, **5**, 133.

Katoh K, Standley DM (2013). MAFFT multiple sequence alignment software version 7: improvements in performance and usability**.** *Molecular Biology and Evolution* **30**: 772-780.

Li, H. and Durbin, R. 2009, Fast and accurate short read alignment with Burrows-Wheeler transform. *Bioinformatics*, **25**, 1754-1760

McKenna A, Hanna M, Banks E, Sivachenko A, Cibulskis K, Kernytsky A, Garimella K, Altshuler D, Gabriel S, Daly M, DePristo MA (2010). The genome analysis toolkit: a papreduce framework for analyzing next-generation DNA sequencing data. *Genome Research* **20***:* 1297-1303.

Neves LG, Davis JM, Barbazuk WB, Kirst M (2013). Whole-exome targeted sequencing of the uncharacterized pine genome. *Plant Journal* **75**: 146-156.

[Page AJ](https://www.ncbi.nlm.nih.gov/pubmed/?term=Page%20AJ%5BAuthor%5D&cauthor=true&cauthor_uid=28348851), [Taylor B](https://www.ncbi.nlm.nih.gov/pubmed/?term=Taylor%20B%5BAuthor%5D&cauthor=true&cauthor_uid=28348851), [Delaney AJ](https://www.ncbi.nlm.nih.gov/pubmed/?term=Delaney%20AJ%5BAuthor%5D&cauthor=true&cauthor_uid=28348851), [Soares J](https://www.ncbi.nlm.nih.gov/pubmed/?term=Soares%20J%5BAuthor%5D&cauthor=true&cauthor_uid=28348851), [Seemann T](https://www.ncbi.nlm.nih.gov/pubmed/?term=Seemann%20T%5BAuthor%5D&cauthor=true&cauthor_uid=28348851), [Keane JA](https://www.ncbi.nlm.nih.gov/pubmed/?term=Keane%20JA%5BAuthor%5D&cauthor=true&cauthor_uid=28348851), [Harris SR](https://www.ncbi.nlm.nih.gov/pubmed/?term=Harris%20SR%5BAuthor%5D&cauthor=true&cauthor_uid=28348851) (2016). *SNP-sites*: rapid efficient extraction of SNPs from multi-FASTA alignments. *Microbiology Genomics* **29**: e000056.

Pennington RT, Prado DE, Pendry CA (2000). Neotropical seasonally dry forests and Quaternary vegetation changes. *Journal of Biogeography* **27**: 261-273.

Prado DE, Gibbs PE (1993). Patterns of species distributions in the dry seasonal forests of South-America. *Annals of the Missouri Botanical Garden* **80**: 902-927.

- Pritchard JK, Stephens M, Donnelly P (2000). Inference of population structure using multilocus genotype data. *Genetics* 155: 945–959.

[Quinlan AR](https://www.ncbi.nlm.nih.gov/pubmed/?term=Quinlan%20AR%5BAuthor%5D&cauthor=true&cauthor_uid=20110278), [Hall IM](https://www.ncbi.nlm.nih.gov/pubmed/?term=Hall%20IM%5BAuthor%5D&cauthor=true&cauthor_uid=20110278) (2010). BEDTools: a flexible suite of utilities for comparing genomic features. [*Bioinformatics*](https://www.ncbi.nlm.nih.gov/pubmed/20110278) **26**: 841-842.

Rambaut A, Grassly NC (1997). Seq-Gen: an application for the Monte Carlo simulation of DNA sequence evolution along phylogenetic trees. *Computer Applications in Bioscience* **13**: 235-238.

Silva-Junior OB, Grattapaglia D (2015). Genome-wide patterns of recombination, linkage disequilibrium and nucleotide diversity from pooled resequencing and single nucleotide polymorphism genotyping unlock the evolutionary history of *Eucalyptus grandis*. *New Phytologist* **208**: 830-845.

Silva-Junior, O B, Grattapaglia D, Novaes E, Collevatti RG (2018a). Genome assembly of the pink ipê (*Handroanthus impetiginosus*, Bignoniaceae), a highly valued, ecologically keystone neotropical timber forest tree. GigaScience 7 (1), gix125.

Silva-Junior OB, Grattapaglia D, Novaes E, Collevatti RG (2018b). Design and evaluation of a sequence capture system for genome-wide SNP genotyping in highly heterozygous plant genomes: a case study with a keystone Neotropical hardwood tree genome. *DNA Research* in press.

Zuur AF, Ieno EN, Walker N, Saveliev AA, Smith GM (2009). Mixed effects models and extensions in ecology with R. In: Gail M, Krickeberg M, Samet JM, Tsiatis A, Wong W, eds. *Statistics for Biology and Health.* New York: Springer-Verlag, pp 261–293.
