# Supplementary material for: A genome-wide scan shows evidence for local adaptation in a widespread keystone Neotropical forest tree
Source: Heredity (Edinb). 2019 Feb 12;123(2):117–37. doi: 10.1038/s41437-019-0188-0 (PMC6781148; doi:10.1038/s41437-019-0188-0)
Supplement: Supplementary file 2 — Supporting Information S1 - Tables [file 41437_2019_188_MOESM2_ESM.doc]

**A genome-wide scan shows evidence for local adaptation in a widespread keystone Neotropical forest tree**

Rosane G. Collevatti, Evandro Novaes, Orzenil Bonfim da Silva-Junior, Lucas Vieira, Matheus S. Lima-Ribeiro, Dario Grattapaglia

**Supporting Information S1 - Tables**

**Table S1.** Sampling locations of the 13 populations of *Handroanthus impetiginosus*.

| Code | Population Locality | Latitude | Longitude |
| --- | --- | --- | --- |
| **ALT** | Altamiro Pacheco Forest Park, GO | S16 32 20.1 | W49 08 17.5 |
| **BAG** | Barra do Garça, MT | S15 07 52.9 | W51 40 09.4 |
| **BRA** | Brasilândia de Minas, MG | S16 45 51.5 | W46 08 36.6 |
| **CAC** | Cáceres, MT | S16 12 33.3 | W57 34 26.0 |
| **MIM** | Mimoso, GO | S15 02 15.5 | W48 09 10.0 |
| **MOC** | Montes Claros, MG | S16 08 57.2 | W43 43 08.9 |
| **NIQ** | Niquelândia, GO | S14 27 13.2 | W48 19 04.7 |
| **PAN** | Januária, MG | S15 30 53.6 | W44 41 32.5 |
| **POF** | Porto Ferro, MA | S6 19 55.5 | W47 13 50.0 |
| **POS** | POS, Posse, GO | S14 03 48.1 | W46 29 09.6 |
| **POT** | Portelândia, GO | S17 17 30.5 | W52 38 20.5 |
| **SEC** | Campo Maior, PI | S4 51 37.4 | W42 03 36.5 |
| **SUM** | Lagoa Santa, MG | S19 32 24.3 | W43 56 02.2 |

**Table S2.** Factorial analysis for climate bioclimatic variables from the WorldClim Global Climate Bioclim database ([www.worldclim.org/bioclim](http://www.worldclim.org/bioclim)) for the 13 populations of *Handroanthus impetiginosus.* Bio1, mean annual temperature; Bio3, isothermality (mean diurnal range /temperature annual range); Bio4, temperature seasonality; Bio5, maximum temperature of warmest month; Bio6, minimum temperature of coldest month; Bio12, annual precipitation; Bio13, precipitation of the wettest month; Bio14, precipitation of driest month; Bio15, precipitation seasonality; Bio16, precipitation of the wettest quarter; Bio17, precipitation of the driest quarter. SS, standard deviation. In bold, variables with higher correlation.

| **Loadings** | **1** | **2** | **3** | **4** |
| --- | --- | --- | --- | --- |
| **Bio1** |  | -0.182 | 0.892 | 0.396 |
| **Bio3** | 0.297 |  | 0.229 | 0.637 |
| **Bio4** | -0.345 |  | -0.222 | **-0.868** |
| **Bio5** |  | -0.268 | **0.931** |  |
| **Bio6** | 0.139 |  | 0.758 | 0.613 |
| **Bio12** | 0.916 | 0.243 |  | 0.229 |
| **Bio13** | 0.931 |  |  | 0.263 |
| **Bio14** |  | 0.944 | -0.208 |  |
| **Bio15** | 0.247 | -0.779 | 0.156 | 0.214 |
| **Bio16** | **0.969** |  |  | 0.204 |
| **Bio17** | 0.192 | **0.950** |  | 0.114 |
| **SS loadings** | 2.985 | 2.588 | 2.434 | 1.916 |
| **Proportion Variation** | 0.271 | 0.235 | 0.221 | 0.174 |
| **Cumulative Variation** | 0.271 | 0.507 | 0.728 | 0.902 |

**Table S3.** Factorial analysis for subsoil (30 – 100 cm) data related to soil fertility from the Harmonized World Soil Database (version 1.2, FAO/IIASA/ISRIC/ISS-CAS/JRC 2009), for the 13 populations of *Handroanthus impetiginosus*. CEC, cationic exchange capacity of clay and soil; BS, soil base saturarion; CaCO3, soil calcium carbonate concentration. In bold, variables with higher correlation

| **Loadings** | **1** | **2** | **3** |
| --- | --- | --- | --- |
| **pH** | 0.366 | 0.621 | 0.631 |
| **CEC CLAY** | **0.738** | 0.543 | 0.252 |
| **CEC SOIL** | 0.676 | 0.124 | 0.241 |
| **BS** | 0.486 | 0.449 | **0.722** |
| **CaCO3** | 0.183 | **0.681** | 0.262 |
| **SS loadings** | 1.405 | 1.361 | 1.109 |
| **Proportion Var** | 0.281 | 0.272 | 0.222 |
| **Cumulative Var** | 0.281 | 0.553 | 0.775 |

**Table S4.** Genetic diversity of the 13 populations of *Handroanthus impetiginosus*, based on 75,838 SNPs. N, number of individuals genotyped; N loci, number of polymorphic loci; *He*, mean expected heterozygosity under Hardy-Weinberg equilibrium; *f*, inbreeding coefficient (* significant, p = 0.032).

| Population | N | N loci | *He* (SD) | *f* |
| --- | --- | --- | --- | --- |
| **ALT** | 5 | 29090 | 0.385(0.339) | 0.296 |
| **BAG** | 10 | 15245 | 0.316(0.379) | -0.097 |
| **BRA** | 12 | 21288 | 0.284(0.390) | -0.099 |
| **CAC** | 11 | 17308 | 0.286(0.390) | -0.015 |
| **MIM** | 11 | 15743 | 0.301(0.406) | -0.395 |
| **MOC** | 12 | 13083 | 0.380(0.387) | -0.359 |
| **NIQ** | 7 | 16556 | 0.325(0.379) | -0.091 |
| **PAN** | 12 | 20163 | 0.285(0.395) | -0.006 |
| **POF** | 5 | 31806 | 0.415(0.358) | -0.511 |
| **POS** | 11 | 17420 | 0.308(0.390) | -0.035 |
| **POT** | 9 | 34859 | 0.349(0.345) | 0.566* |
| **SEC** | 12 | 15376 | 0.308(0.394) | -0.048 |
| **SUM** | 11 | 30836 | 0.269(0.379) | -0.104 |
| **Mean** | 9.8 |  | 0.324 |  |
| **SD** | 2.6 |  | 0.379 |  |
| **Total** | 128 |  | 0.169(0.138) | -0.135 |

**Table S5.** Proportion of membership (*Q*) of each pre-defined population of *Handroanthus impetiginosus* in Brazil in each of the 4 clusters inferred by Bayesian analyses implemented in STRUCTURE software. N, number of individuals sampled in each population. In bold, Q ≥ 0.100.

|  | **Inferred Cluster** | | | |  |
| --- | --- | --- | --- | --- | --- |
| **Population** | **1** | **2** | **3** | **4** | **N** |
| **ALT** | 0.004 | 0.203 | **0.790** | 0.003 | 5 |
| **BAG** | 0.007 | **0.990** | 0.000 | 0.003 | 10 |
| **BRA** | **0.881** | **0.109** | 0.001 | 0.010 | 12 |
| **CAC** | 0.004 | **0.993** | 0.000 | 0.003 | 11 |
| **MIM** | 0.045 | **0.944** | 0.002 | 0.009 | 11 |
| **MOC** | 0.000 | 0.000 | **0.999** | 0.000 | 12 |
| **NIQ** | 0.083 | **0.903** | 0.001 | 0.013 | 7 |
| **PAN** | **0.919** | 0.046 | 0.000 | 0.034 | 12 |
| **POF** | 0.081 | **0.269** | **0.391** | **0.259** | 5 |
| **POS** | **0.947** | 0.038 | 0.000 | 0.015 | 11 |
| **POT** | 0.024 | **0.739** | **0.222** | 0.014 | 9 |
| **SEC** | 0.006 | 0.005 | 0.000 | **0.988** | 12 |
| **SUM** | 0.047 | 0.045 | **0.896** | 0.011 | 11 |

**Table S6**. Comparison of the four demographical scenario models in retrieving the genetic diversity (*He*) observed for *Handroanthus impetiginosus,* obtained from 2,000 simulations using the software *fastsimcoal2*. *ΔAIC,* the difference of *AICw* between each model and the best model; *AICw,* Akaike Information Criterion (*AIC*) weights; *P*, two-tailed probability of not rejecting the model. PLAH, Pleistocene Arc Hypothesis, i.e. an expansion throughout the Central and Southwest Brazil; PPPH, Amazon SDTF Hypothesis, i.e. a westward range shift, toward the Amazon Basin; Both, PLAH+PPPH, i.e. a prediction for the past distribution as expected by Pleistocene Arc and Amazon SDTF hypotheses, resulting in an expansion throughout the Central and Southwest Brazil and also towards the interior of Amazon Basin; Range Retraction, i.e. a retraction in geographical range in Central Brazil but without range shift. Mean and SD are the *He* average and standard deviation across all simulations.

| **Models** | ***ΔAIC*** | ***AICw*** | ***P*** | ***Mean*** | ***SD*** |
| --- | --- | --- | --- | --- | --- |
| **PLAH** | 1.25 | 0.29 | 0.729 | 0.107 | 0.180 |
| **PPPH** | 3.87 | 0.08 | 0.148 | 0.064 | 0.138 |
| **BOTH** | 0.00 | 0.54 | 0.789 | 0.097 | 0.154 |
| **Retraction** | 3.42 | 0.09 | 0.179 | 0.052 | 0.150 |

**Table S7**. Environmental variables used in Bayenv2 and co-kriging analyses, for each sampled population. Bio4, temperature seasonality; Bio5, maximum temperature of warmest month; Bio16, precipitation of wettest quarter; Bio17 precipitation of driest quarter; CEC Clay, subsoil clay cationic exchange capacity; BS, subsoil base saturation; CACO_3_, subsoil clay calcium carbonate. SD, standard variation.

| **Population** | **Latitude** | **Longitude** | **Bio 4** | **Bio 5** | **Bio 16** | **Bio 17** | **CEC Clay** | **BS** | **CACO3** |
| --- | --- | --- | --- | --- | --- | --- | --- | --- | --- |
| **ALT** | -16.589 | -49.182 | 244.667 | 31.110 | 665.261 | 9.065 | 7.252 | 36.915 | 0.00146 |
| **BAG** | -15.264 | -51.693 | 195.106 | 33.443 | 736.615 | 3.544 | 13.616 | 25.166 | 0.00001 |
| **BRA** | -16.893 | -46.235 | 244.357 | 29.641 | 476.772 | 19.258 | 8.124 | 34.614 | 0.00001 |
| **CAC** | -16.293 | -57.639 | 166.778 | 35.721 | 621.733 | 8.433 | 18.203 | 33.173 | 0.00001 |
| **MIM** | -15.076 | -48.178 | 224.783 | 30.655 | 641.825 | 10.490 | 9.361 | 14.063 | 0.00001 |
| **MOC** | -16.292 | -43.741 | 268.646 | 29.368 | 327.597 | 39.348 | 15.266 | 73.987 | 0.52745 |
| **NIQ** | -14.487 | -48.330 | 199.168 | 31.842 | 688.965 | 7.951 | 10.294 | 23.060 | 0.00001 |
| **PAN** | -15.649 | -44.774 | 249.169 | 30.488 | 378.888 | 25.076 | 10.516 | 33.545 | 0.00014 |
| **POF** | -6.471 | -47.356 | 193.060 | 34.503 | 1398.909 | 27.280 | 9.572 | 29.595 | 0.00001 |
| **POS** | -14.184 | -46.510 | 211.682 | 31.070 | 582.975 | 13.881 | 11.574 | 25.109 | 0.00001 |
| **POT** | -17.368 | -52.690 | 214.011 | 31.778 | 804.264 | 7.548 | 6.400 | 21.600 | 0.04000 |
| **SEC** | -4.954 | -42.151 | 135.458 | 33.897 | 1277.112 | 53.092 | 9.083 | 25.127 | 0.00372 |
| **SUM** | -19.601 | -43.939 | 285.117 | 26.433 | 475.194 | 65.140 | 10.336 | 34.129 | 0.01425 |
| **Mean** |  |  | 217.846 | 31.534 | 698.162 | 22.316 | 10.738 | 31.545 | 0.04516 |
| **SD** |  |  | 0.190 | 0.078 | 0.453 | 0.865 | 0.306 | 0.454 | 3.21886 |
