# Supplementary material for: A genome-wide scan shows evidence for local adaptation in a widespread keystone Neotropical forest tree
Source: Heredity (Edinb). 2019 Feb 12;123(2):117–37. doi: 10.1038/s41437-019-0188-0 (PMC6781148; doi:10.1038/s41437-019-0188-0)
Supplement: Supplementary file 4 — Supporting Information S3 Figures [file 41437_2019_188_MOESM4_ESM.doc]

**A genome-wide scan shows evidence for local adaptation in a widespread keystone Neotropical forest tree**

Rosane G. Collevatti, Evandro Novaes, Orzenil Bonfim da Silva-Junior, Lucas Vieira, Matheus S. Lima-Ribeiro, Dario Grattapaglia

**Supporting Information S3 - Figures**


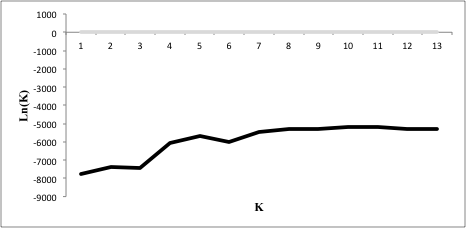


**(a)**


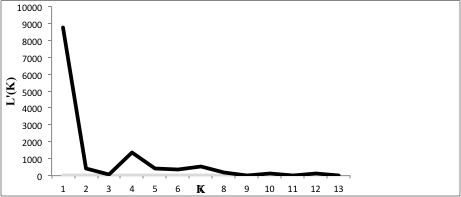


**(b)**


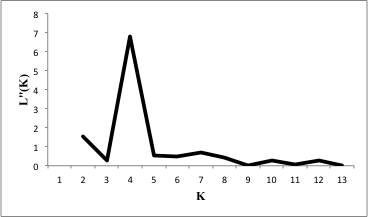


**(c)**

**Fig. S1.** Posterior probability graphs of the Bayesian clustering simulation implemented in the software STRUCTURE 2.3.3 (Pritchard et al. 2000) based on 200 putative neutral SNPs of *Handroanthus impetiginosus*. (**a**) Log of the posterior probability of data [L(D|K)] as a function of K averaged over 5 independent runs. (**b**) Differences between the log of the posterior probability of the data {[L(D|K)_n_ - [L(D|K)_n-1_ } as a function of K averaged over 5 independent runs and standardized by K variance. (**c**) Module of the differences between the log of the posterior probability of the data |{[L(D|K)_n_ as- [L(D|K)_n-1_ }| / as a function of K averaged over 5 independent runs and standardized by K variance.

**
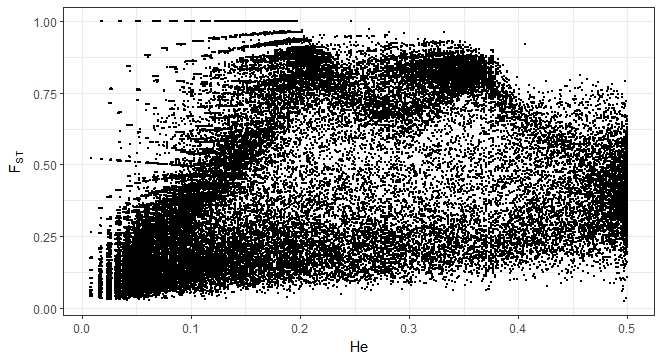
**

**Fig. S2.** Detection of loci under selection based on OUTFLANK and on 75,838 SNPs of *Handroanthus impetiginosus*. The analysis shows no outlier loci.

**
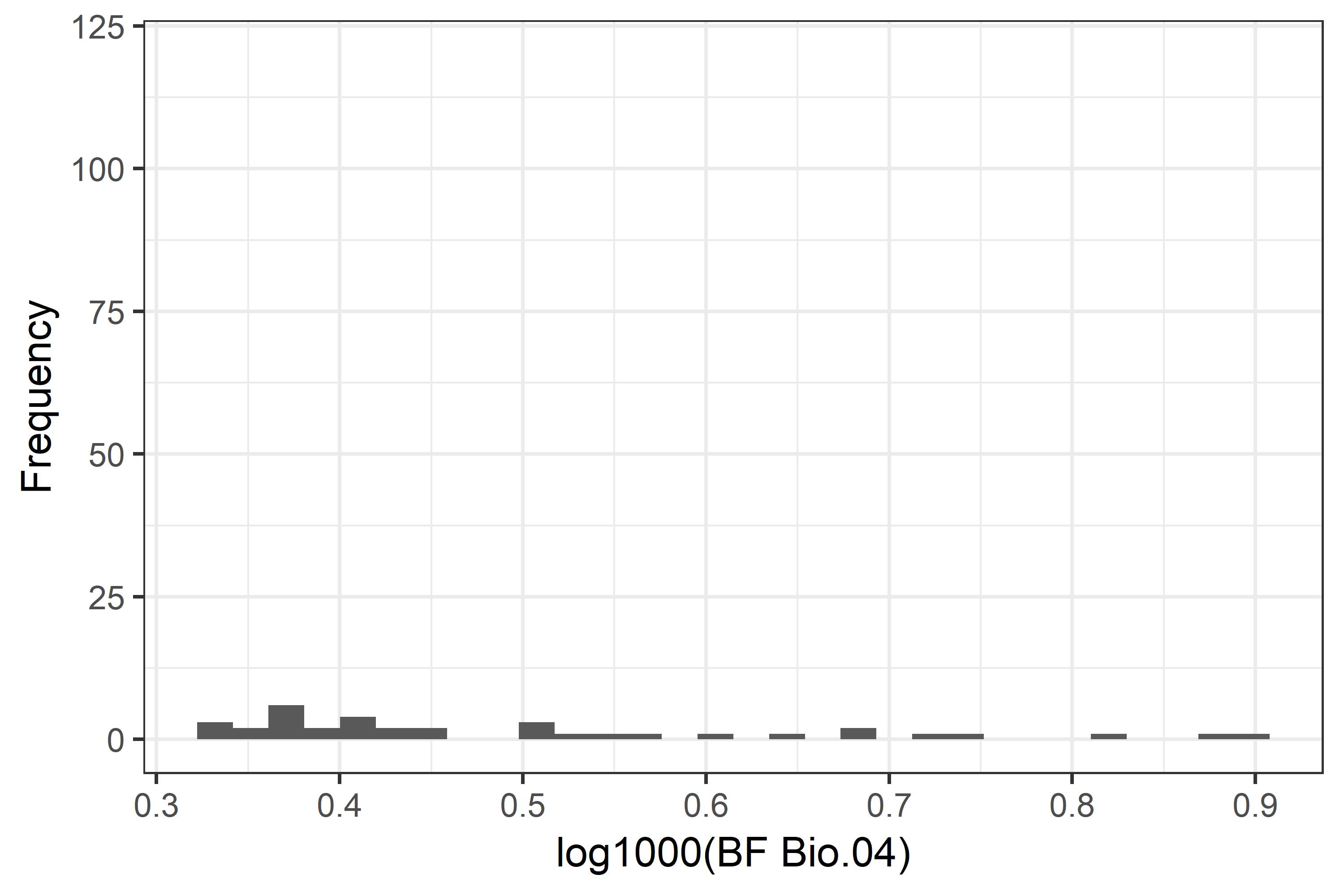
**

**
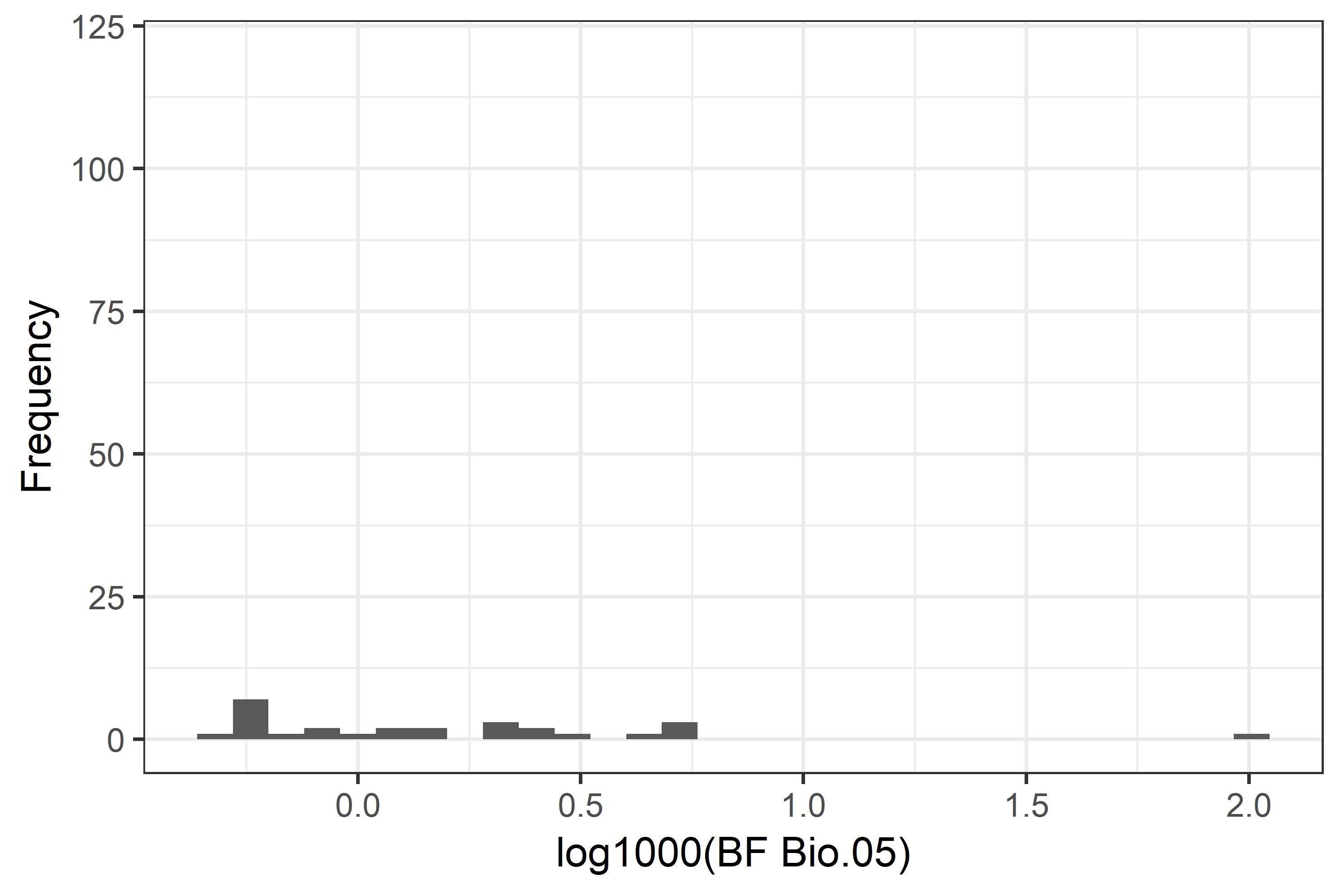
**

**
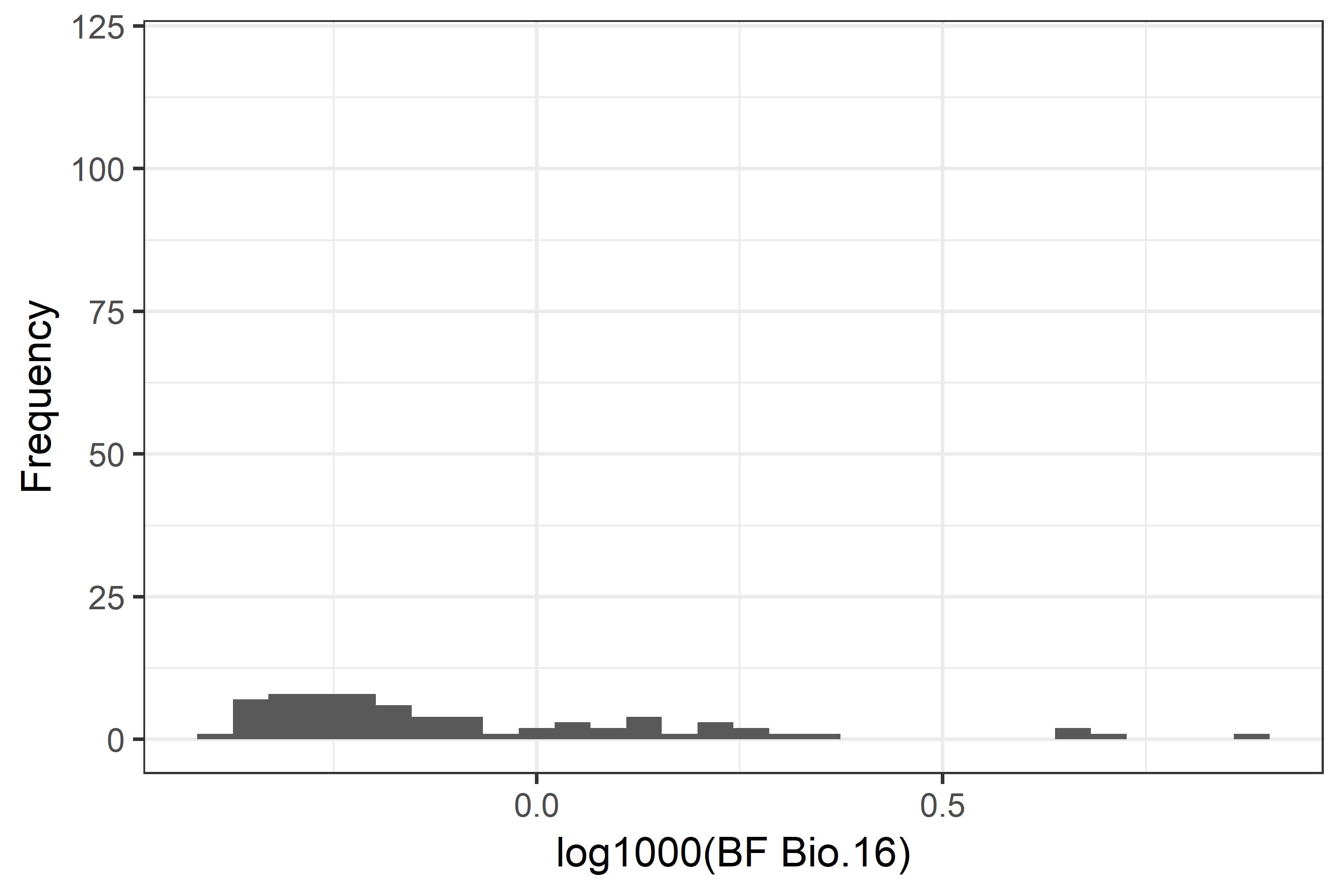
**

**
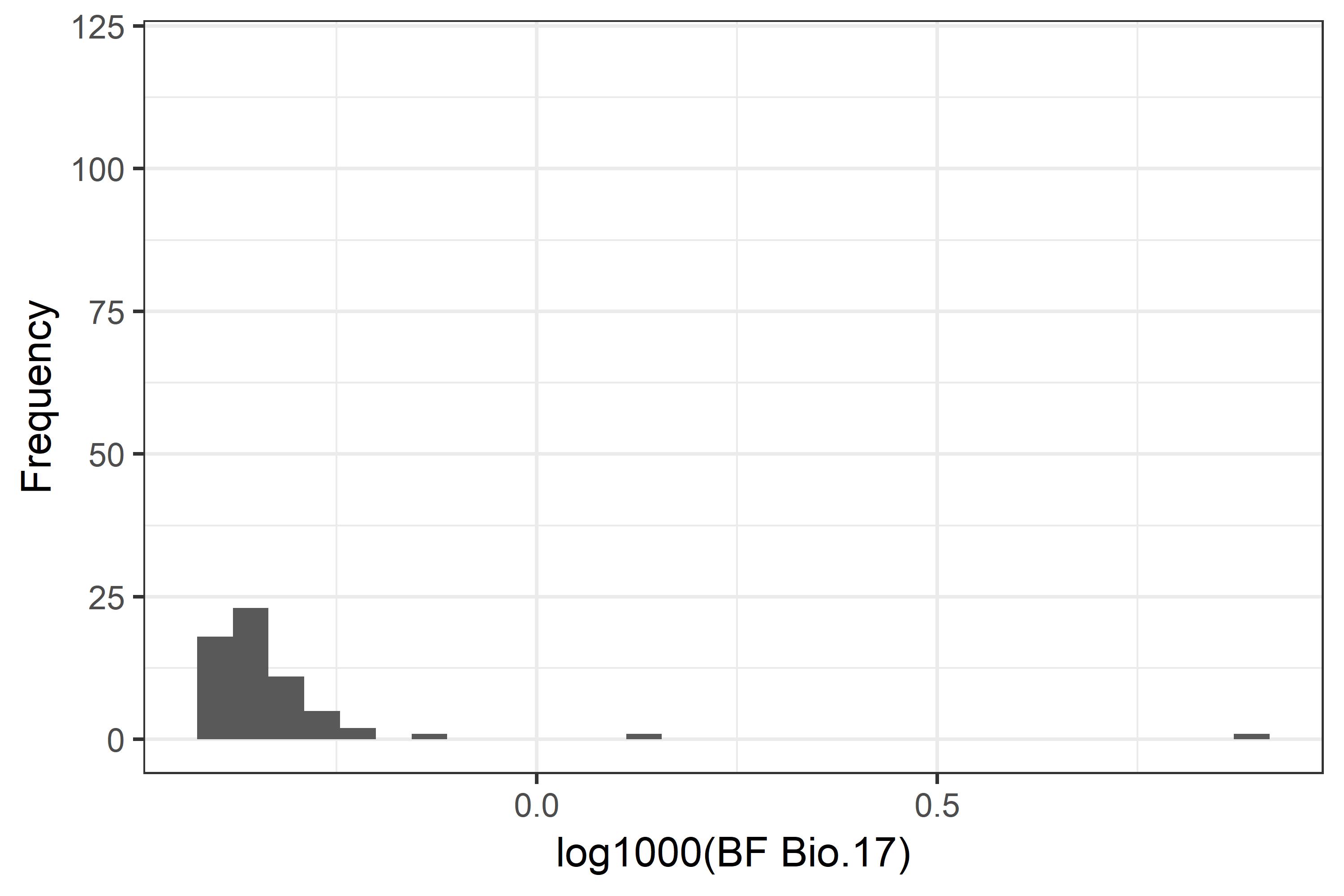
**

**
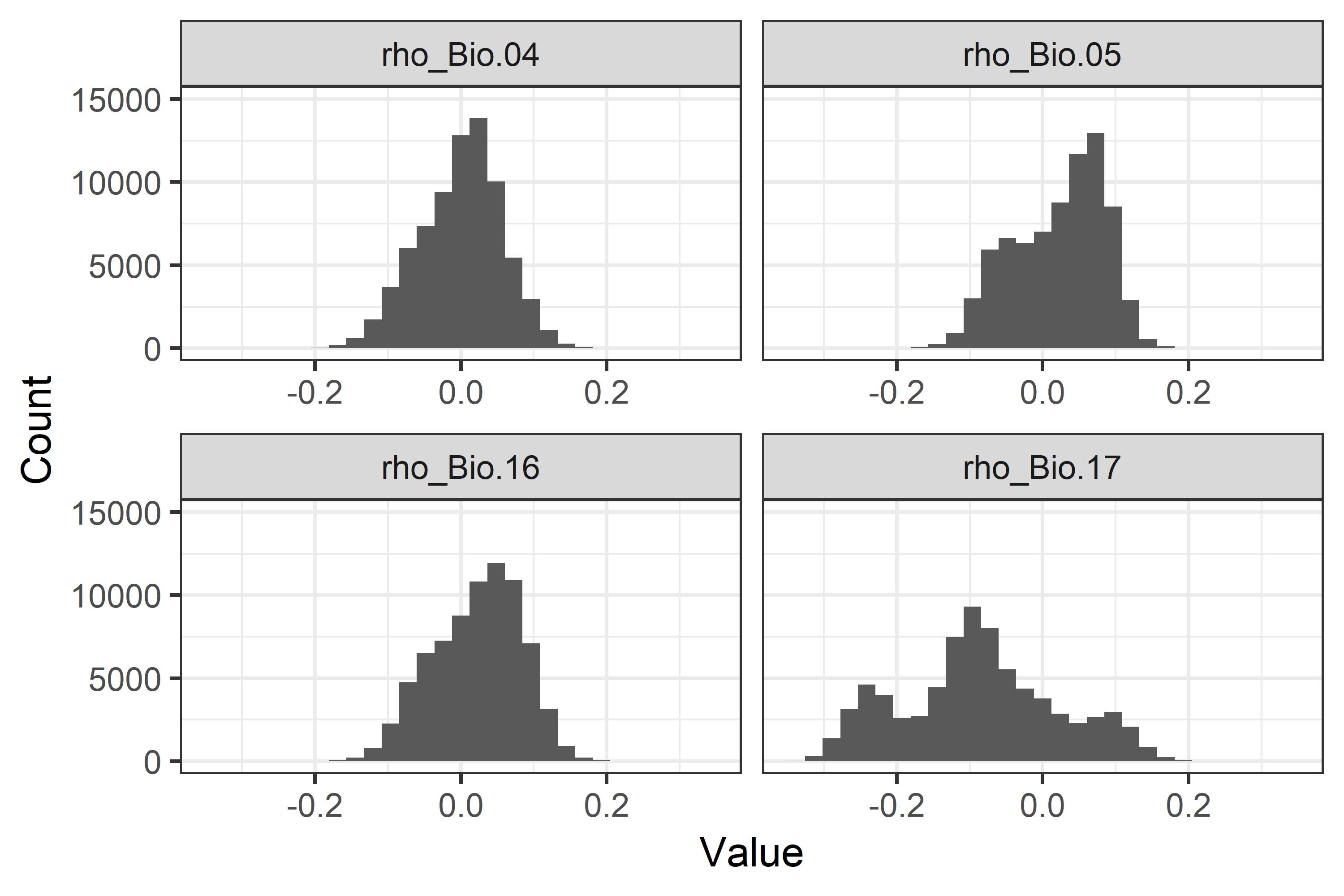
**

**Fig. S3.** Frequency distribution of Bayes factor (BF) and Spearman’s correlation (rho) based on 75,838 SNPs of *Handroanthus impetiginosus* analysed with Bayenv2 software for climatic variables. Bio4, temperature seasonality; Bio5, maximum temperature of warmest month; Bio16, precipitation of wettest quarter; Bio17 precipitation of driest quarter.


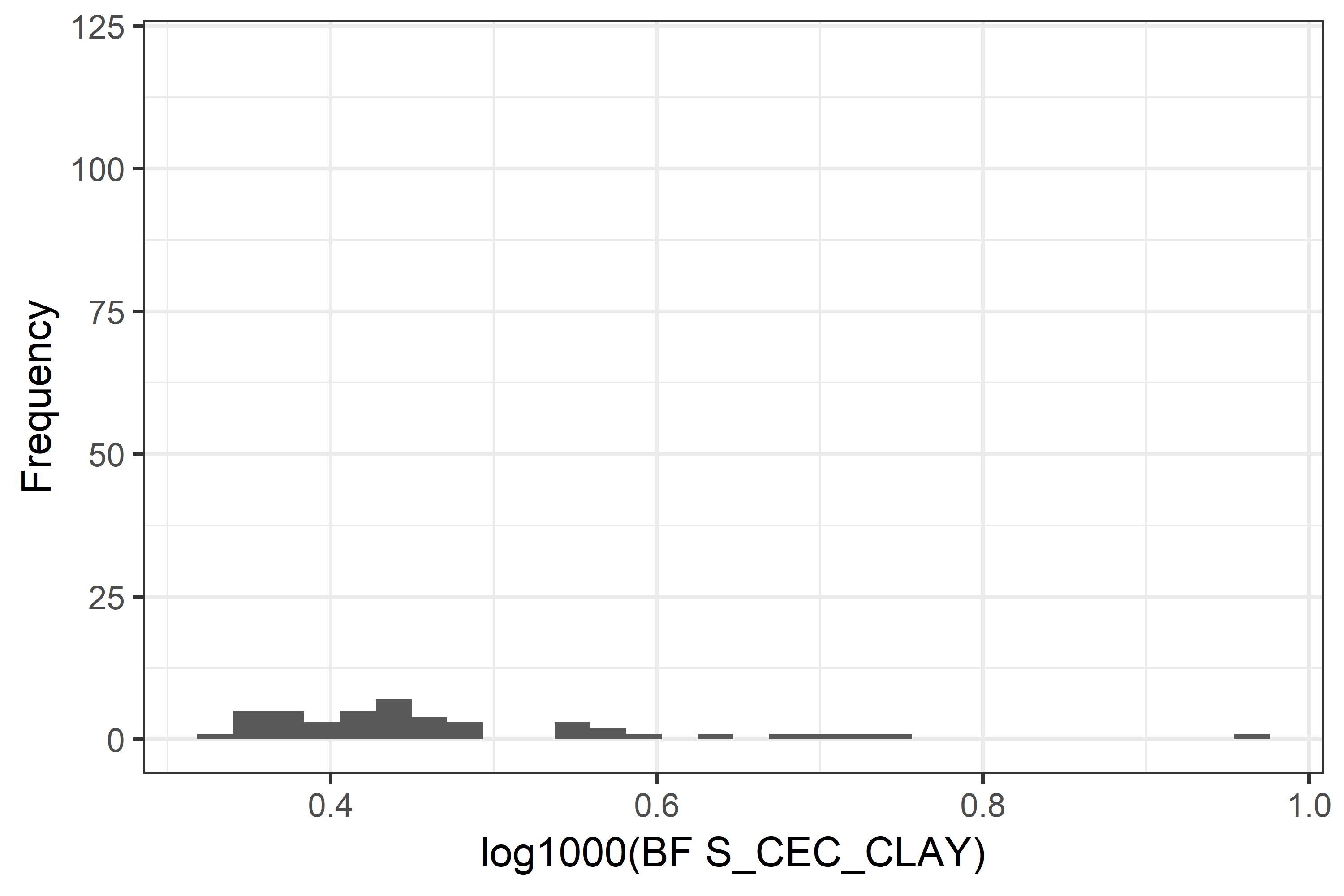


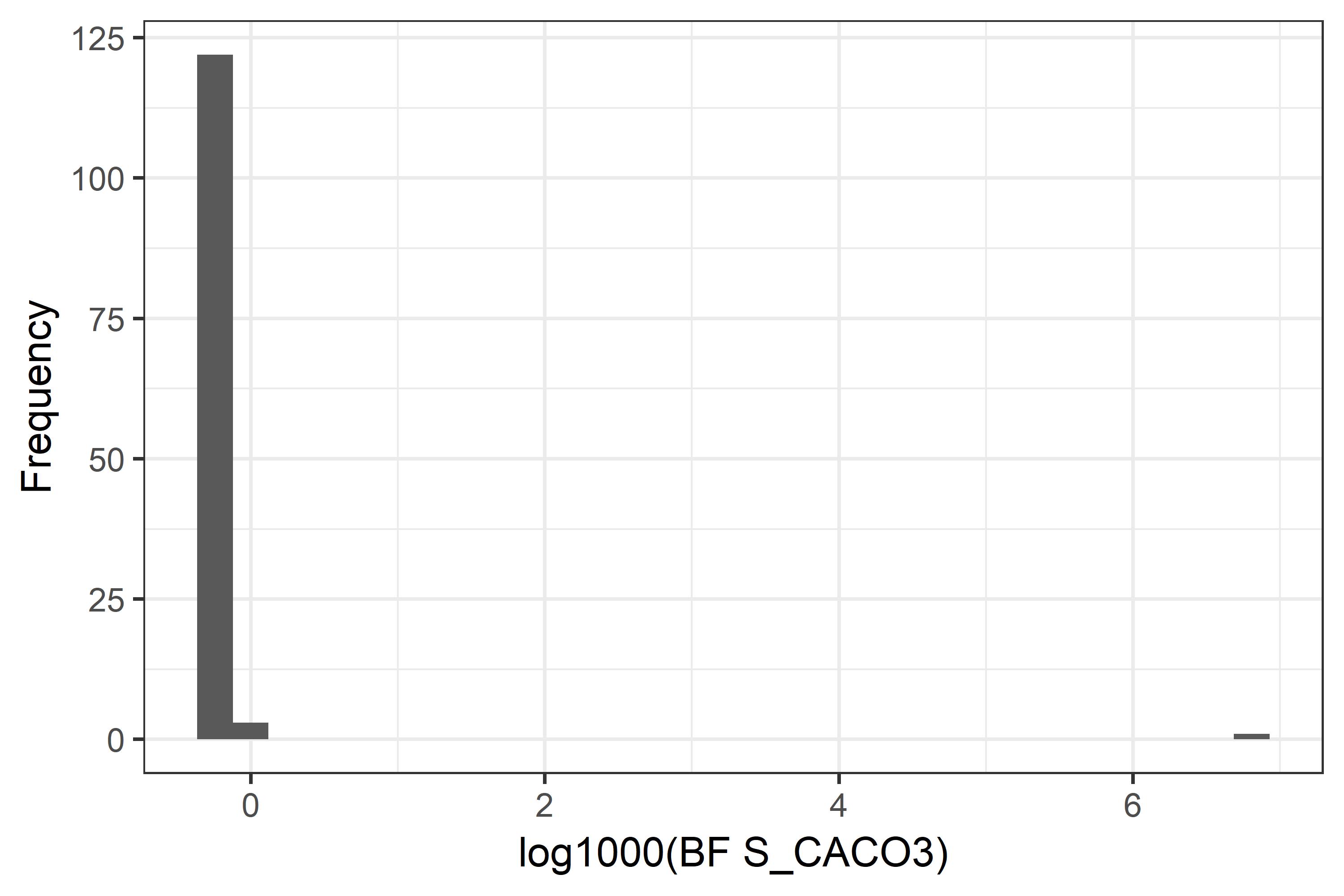


**
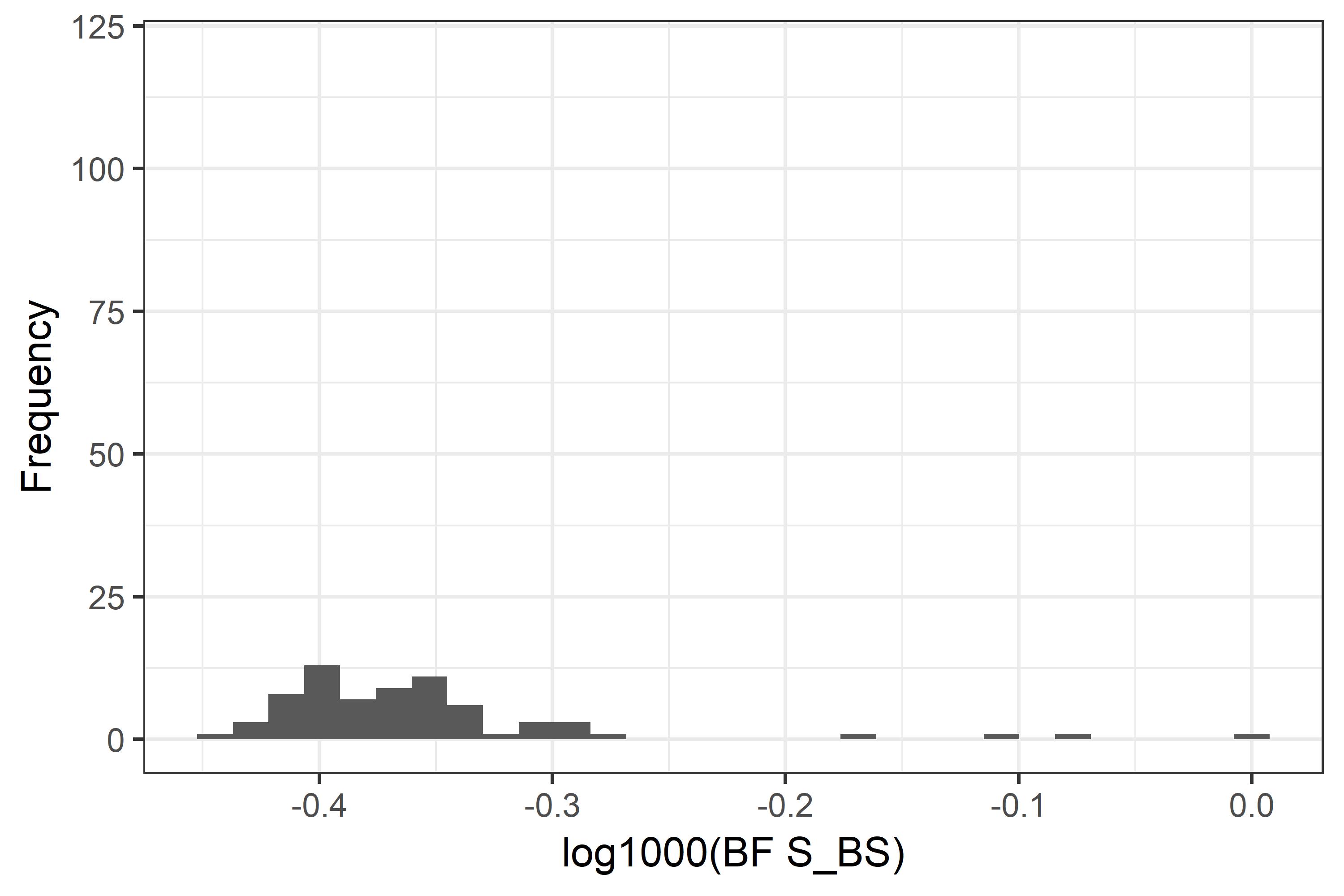
**

**
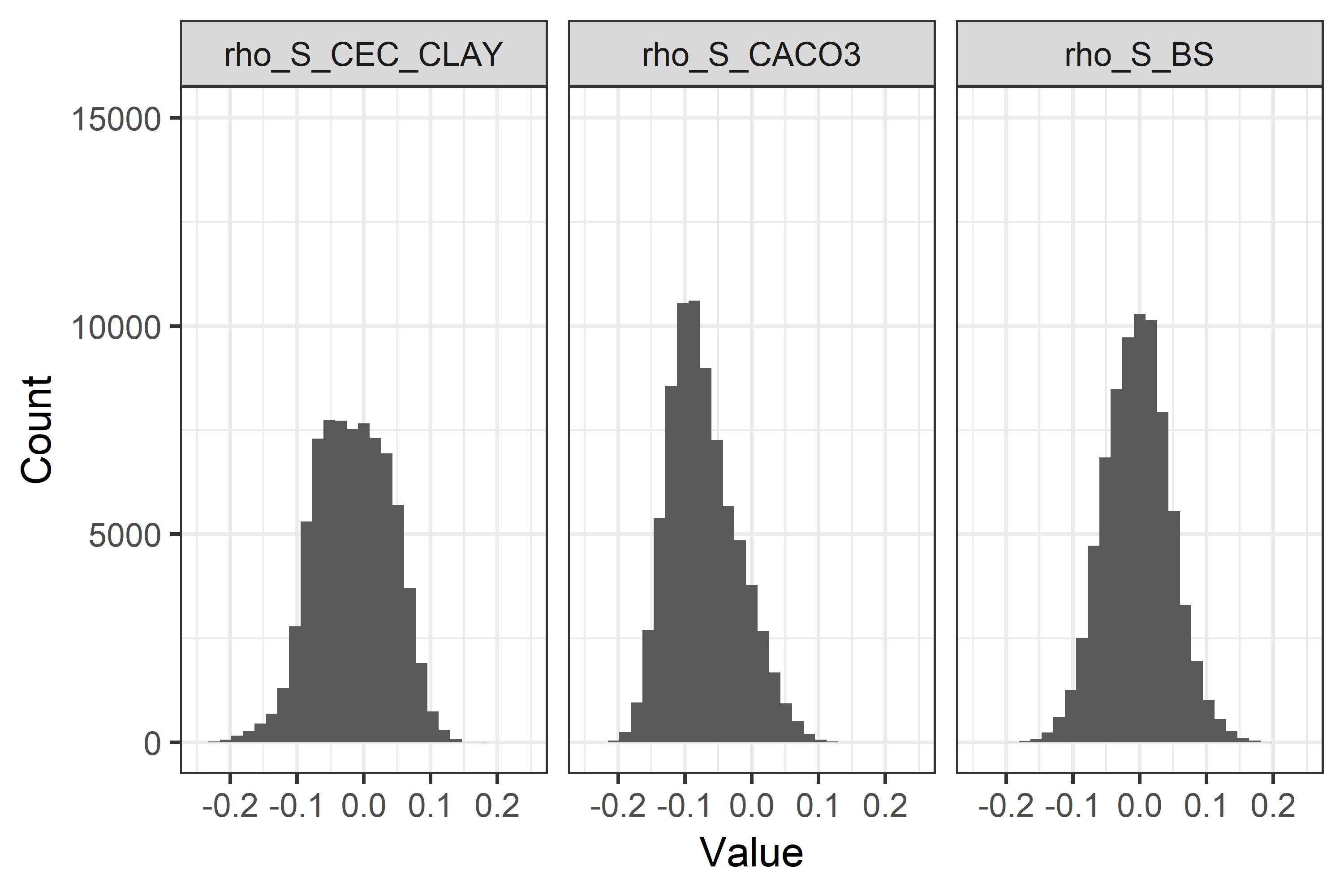
**

**Fig. S4.** Frequency distribution of Bayes factor (BF) and Spearman’s correlation (rho) based on 75,838 SNPs of *Handroanthus impetiginosus* analysed with Bayenv2 software for soil variables. CEC Clay, subsoil clay cationic exchange capacity; CACO_3_, subsoil clay calcium carbonate; BS, subsoil base saturation


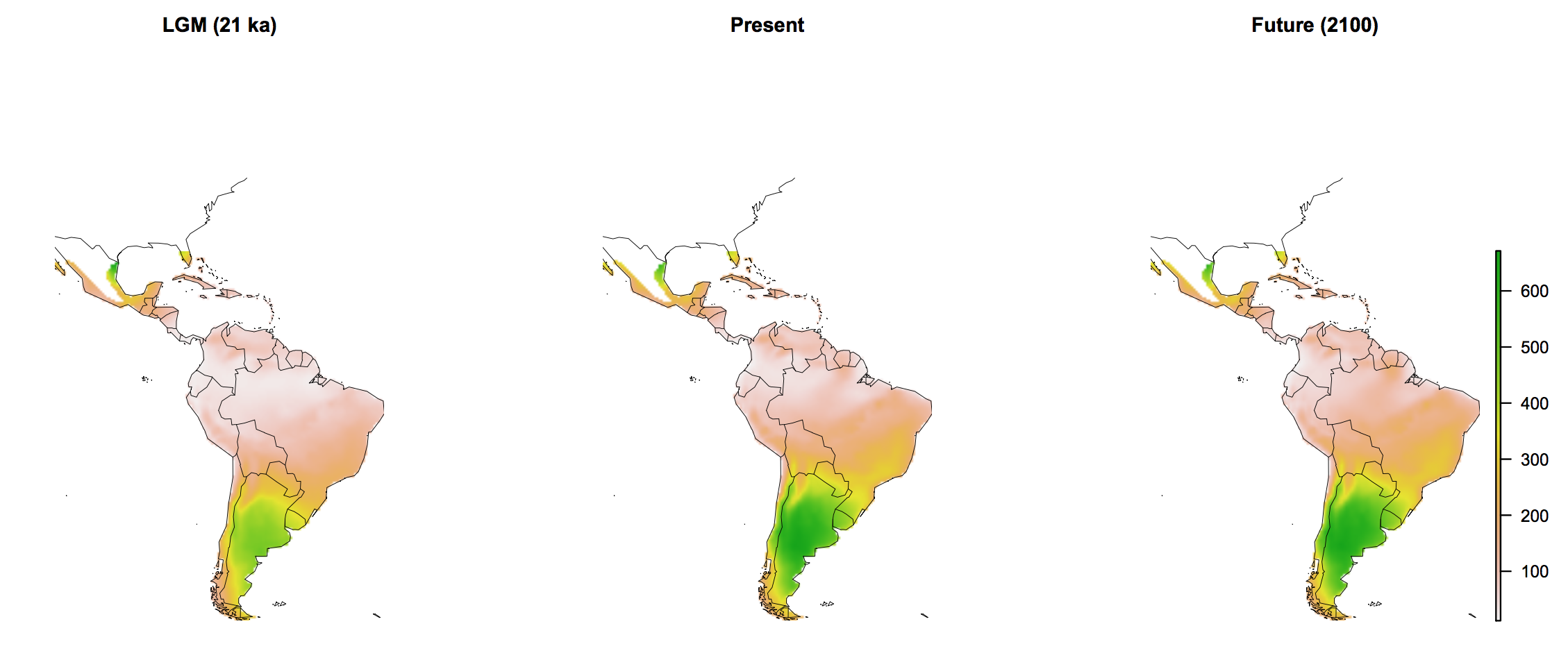


Bio4


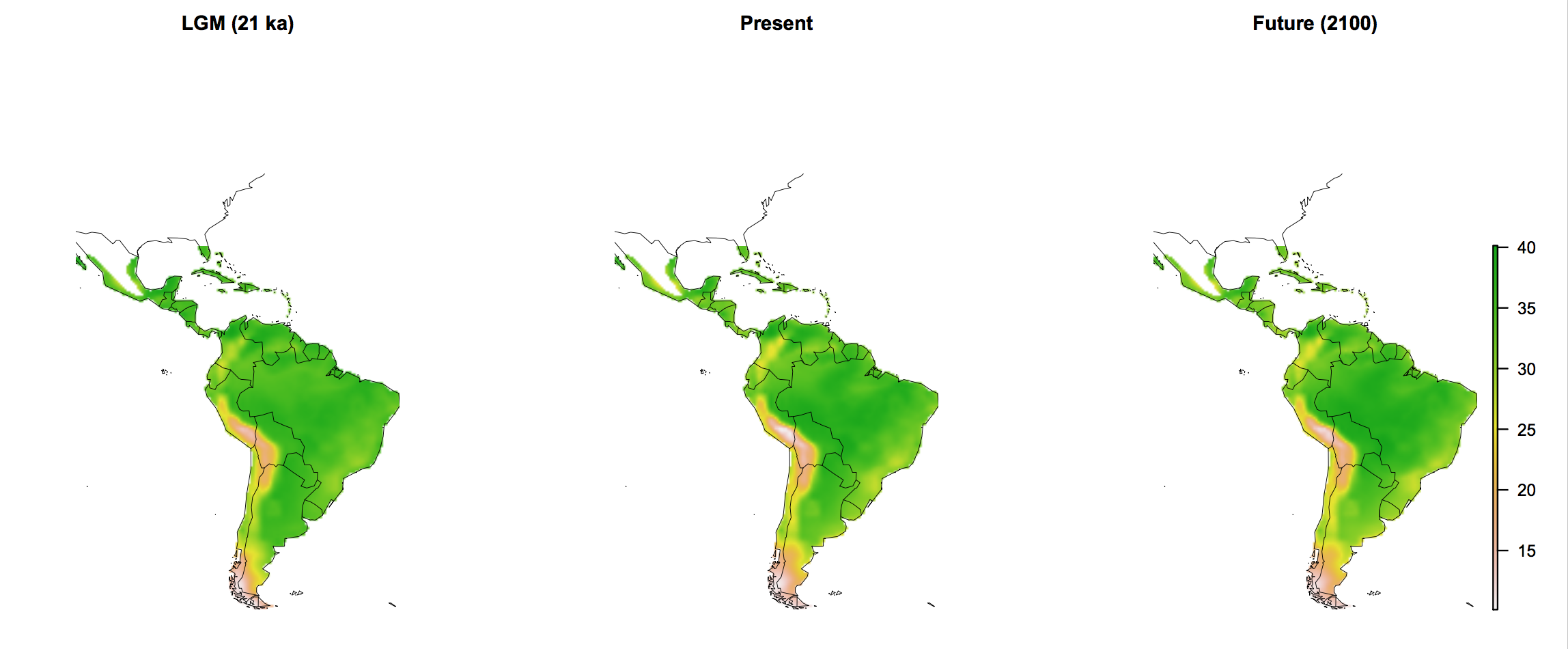


Bio5


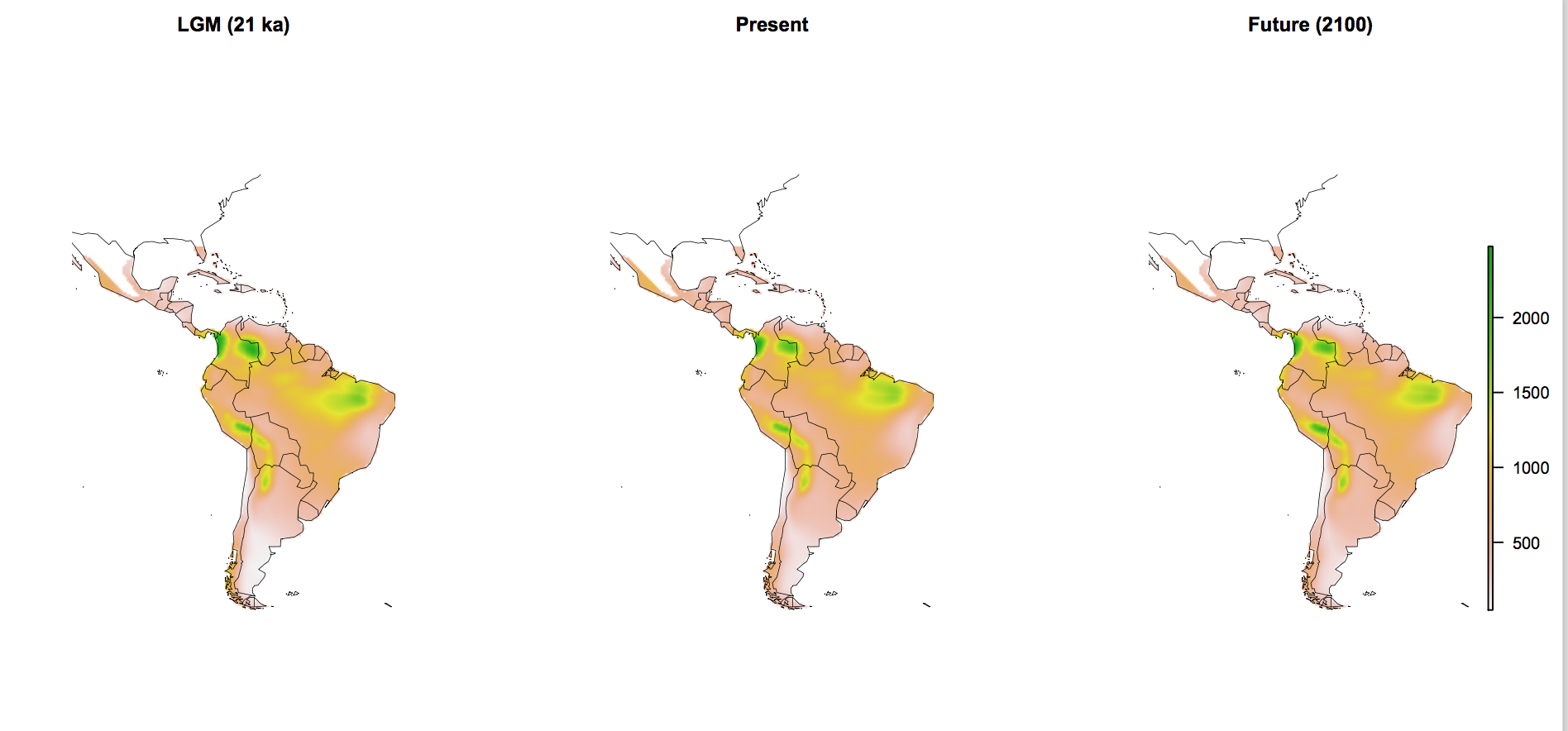


Bio16


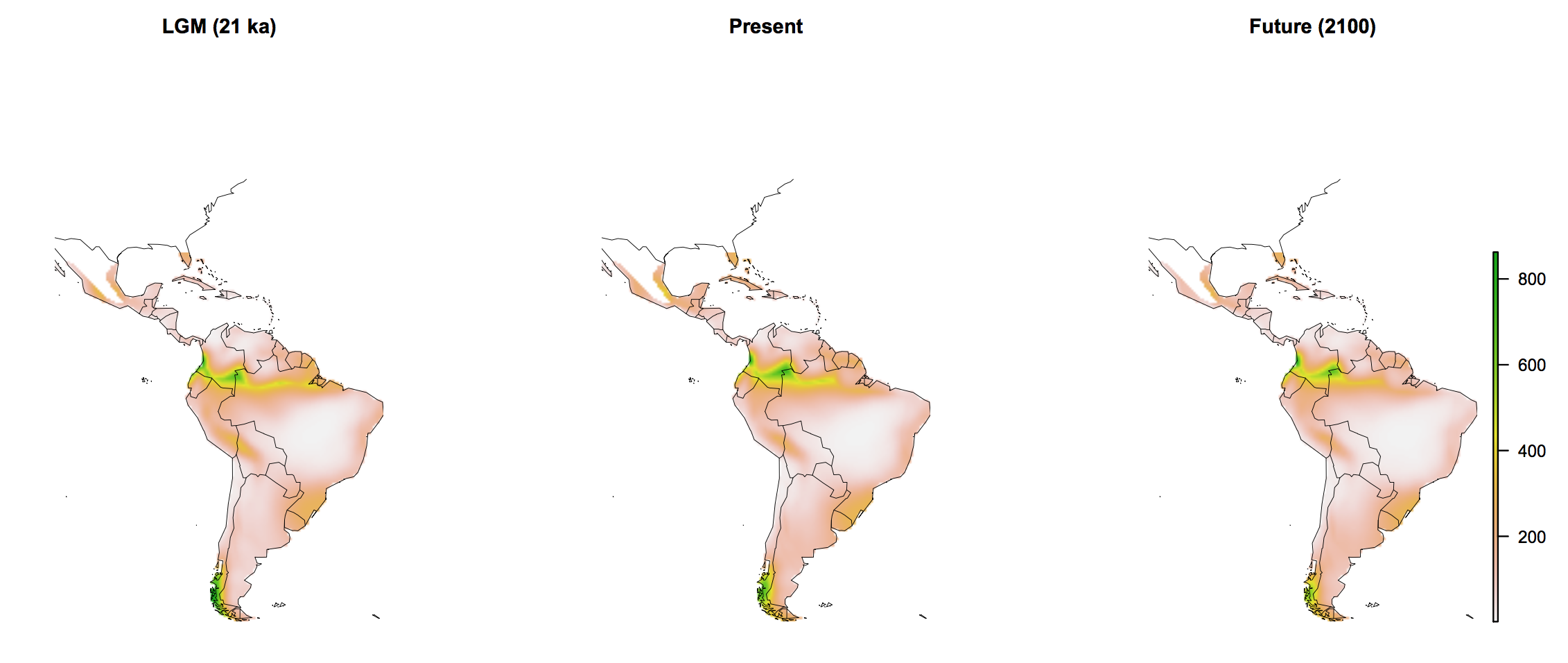


Bio 17

**Fig. S5.** Geographical space of climatic variables in Neotropics during the LGM (21 ka), present-day (0 ka) and the end of the century (2100) for the four bioclimatic variables used for Bayenv2 analysis and co-kriging: Bio4 (temperature seasonality), Bio5 (maximum temperature of warmest month), Bio16 (precipitation of the wettest quarter) and Bio17 (precipitation of the driest quarter).


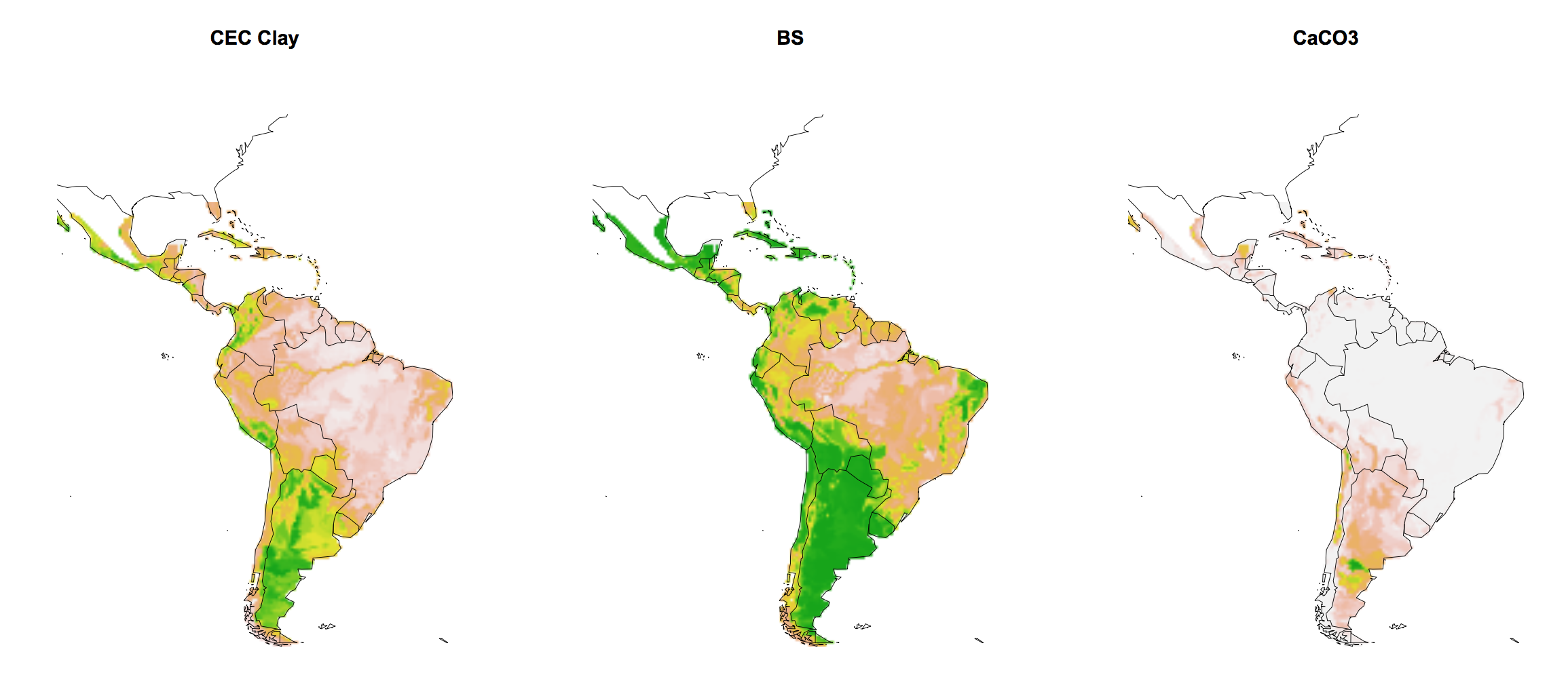


**Fig. S6.** Geographical space of soil variables in Neotropics used for Bayenv2 analysis and co-kriging: clay cationic exchange capacity (CEC Clay), CaCO3 concentration, and base saturation (BS).


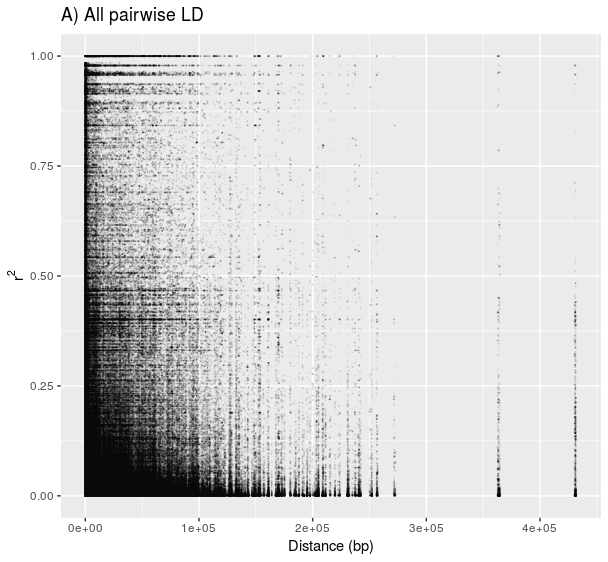


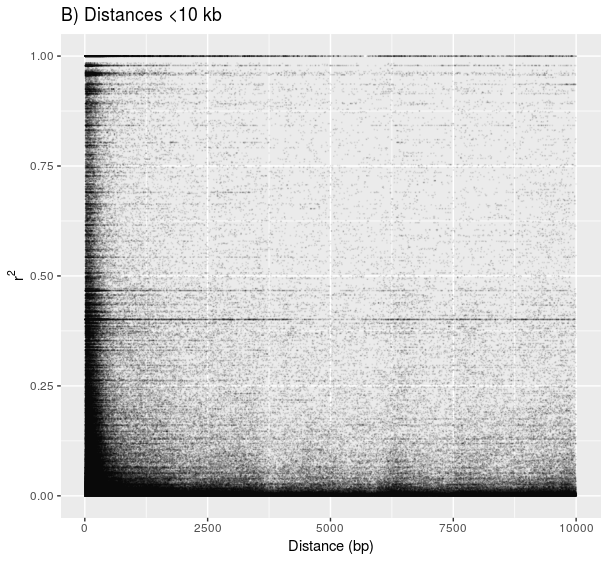


**Fig. S7.** LD decay among all pairwise SNPs (A) and among SNPs at distances shorter than 10 Kb (B). Only SNPs located at scaffolds longer than 50 Kb with at least 15 SNPs were included in the analyses. LD was estimated with *VCFtools* (Danece*k et* al. 2011) after phasing SNPs with fastPHASE 1.4 (Scheet & Stephens 2006, 2008). Plot was performed with ggplot2 package of R, using 95% transparency (alpha = 0.05) for visualization of dot density.

**Reference**

Danecek P, Auton A, Abecasis G, Albers CA, Banks E, DePristo MA, Handsaker B, Lunter G, Marth G, Sherry S, McVean G, Durbin R, and 1000 Genomes Project Analysis Group. 2011. The variant call format and VCFtools. *Bioinformatics (Oxford, England)* **27**: 2156–8

Scheet P, Stephens M. 2006. A Fast and Flexible Statistical Model for Large-Scale Population Genotype Data: Applications to Inferring Missing Genotypes and Haplotypic Phase. *American Journal of Human Genetics* **78**: 629–644.

Scheet P, Stephens M. 2008. Linkage Disequilibrium-based Quality Control for Large-Scale Genetic Studies. *PLoS Genetics* **4**: e1000147.
